# Canonical and single-cell Hi-C reveal distinct chromatin interaction sub-networks of mammalian transcription factors

**DOI:** 10.1186/s13059-018-1558-2

**Published:** 2018-10-25

**Authors:** Xiaoyan Ma, Daphne Ezer, Boris Adryan, Tim J. Stevens

**Affiliations:** 1 Department of Genetics, University of Cambridge, Downing Street, Cambridge, CB2 3EH UK; 2 The Alan Turing Institute for Data Science, British Library, 96 Euston Rd, Kings Cross, London, NW1 2DB UK; 3 Department of Statistics, University of Warwick, Coventry, CV4 7AL UK; 4 Merck KGaA, Chief Digital Office, 64293 Darmstadt, Germany; 5 MRC Laboratory of Molecular Biology, Cambridge Biomedical Campus, Francis Crick Avenue, Cambridge, CB2 0QH UK

**Keywords:** Transcription factor, Genome structure, Nuclear organization, Hi-C, Chromatin conformational capture, Chromosome compartment, Proximity network

## Abstract

**Background:**

Transcription factor (TF) binding to regulatory DNA sites is a key determinant of cell identity within multi-cellular organisms and has been studied extensively in relation to site affinity and chromatin modifications. There has been a strong focus on the inference of TF-gene regulatory networks and TF-TF physical interaction networks. Here, we present a third type of TF network, the spatial network of co-localized TF binding sites within the three-dimensional genome.

**Results:**

Using published canonical Hi-C data and single-cell genome structures, we assess the spatial proximity of a genome-wide array of potential TF-TF co-localizations in human and mouse cell lines. For individual TFs, the abundance of occupied binding sites shows a positive correspondence with their clustering in three dimensions, and this is especially apparent for weak TF binding sites and at enhancer regions. An analysis between different TF proteins identifies significantly proximal pairs, which are enriched in reported physical interactions. Furthermore, clustering of different TFs based on proximity enrichment identifies two partially segregated co-localization sub-networks, involving different TFs in different cell types. Using data from both human lymphoblastoid cells and mouse embryonic stem cells, we find that these sub-networks are enriched within, but not exclusive to, different chromosome sub-compartments that have been identified previously in Hi-C data.

**Conclusions:**

This suggests that the association of TFs within spatial networks is closely coupled to gene regulatory networks. This applies to both differentiated and undifferentiated cells and is a potential causal link between lineage-specific TF binding and chromosome sub-compartment segregation.

**Electronic supplementary material:**

The online version of this article (10.1186/s13059-018-1558-2) contains supplementary material, which is available to authorized users.

## Background

Sequence-specific transcription factors (TFs) are regulatory proteins that bind DNA sequence motifs to activate or repress target genes [1–6]. In multi-cellular organisms, while there are many universal TFs that act within a wide variety of cell types, others are only active in a subset. This is especially important for the establishment and maintenance of linage-specific gene expression patterns and hence for defining cell identity [5, 6]. Consequently, cell-specific TFs are often used as a marker for those lineages [6–8]. ChIP-seq experiments have been extensively employed in various cell types to show where TFs bind in a genome-wide sequence context. Also, it has been shown that ChIP-seq peaks for some TFs expressed in the same cell type tend to overlap with each other [9–11], due to both physical interactions and/or closely coupled gene regulation.

A genome, however, has spatial dimensionalities of structure beyond its linear DNA sequence which could affect and/or be affected by TF binding. Furthermore, the rate of TF binding might be affected by the non-uniform concentrations of TFs within the nucleus [12]. Various studies have probed the 3D distribution of TF binding in the nucleus, both looking at clustering of a particular TF (homotypic) and at the association of different, non-identical TFs (heterotypic) [13–16]. For instance, using single molecule tracking and FCS, fluorescently tagged Sox2 has been shown to self-associate in the nucleus more often than expected by chance [15]. Also, live imaging of c-Fos and c-Jun proteins, which are known to interact, has revealed that they co-localize and co-bind within the nucleus [16]. Furthermore, based on simulations of binding dynamics, it has been predicted that spatial clustering of TF sites of the same type could facilitate TF binding [12, 17]. As illustrated by Sox2, for which it is predicted that clustering is important for increasing association rates [15], there are hints that purely spatial binding site clustering really can influence in vivo TF binding. However, it is not known how any predicted mechanical influence [17–19] from TF association (homotypic and/or heterotypic) varies with the specific TF protein and regulatory context, e.g., as indicated by histone marks.

Overall, such studies suggest that 3D genome organization and TF-TF association might be closely related, but these have only focused on a small number of TFs. In part, this is because concurrently visualising multiple TF localizations using microscopy is restricted to a small number of TFs, due to limited florescence channels and the requirement for tagged proteins. Also, although fluorescence techniques like DNA-FISH can locate specific loci, microscopy does not routinely identify genomic sequence positions. However, chromosome conformation capture techniques such as 3C, 4C, 5C, and Hi-C have developed significantly in recent years and give an alternative means of probing chromatin 3D relationships [20–25]. These techniques generate DNA-DNA proximity information via sequence ligation and can be used to detect and quantify spatial genomic interactions, such as loops and enhancers [20, 26, 27]. For example, the α-globin promoter and its distal enhancer have been shown to be spatially adjacent, via chromosome looping, when the gene is activated [28, 29]. The Hi-C method, which detects chromatin contacts on a genome-wide scale, potentially allows all active TF binding sites to be studied concurrently [25, 30, 31]. However, the extent to which TF interaction networks in general are also spatial chromatin networks, involving co-localization of TFs’ target binding sites, remains unclear.

Genome-wide Hi-C contact maps have revealed that chromosomes are segmented into regions where contacts occur more frequently within those regions than between them. At different size scales, these regions have led to the delineation of various compartments, topologically associating domains (TADs) and loop domains [24, 26, 32–35], and these may be further classified into segregated categories. For example, at the megabase scale, Hi-C contact maps show partitioning into open and closed chromatin, corresponding to the so-called A and B compartments [25, 32] which single-cell Hi-C genome structures show to be a consistent partitioning in the nuclear volume [36]. With the availability of high-resolution Hi-C maps at a kilobase scale, the A and B compartments have been further partitioned based on distinct patterns of long-range contacts. For instance, the inter-chromosome contact map of human lymphoblastoid (GM12878) cells can be sub-divided into at least six different sub-compartments [24], two of which are enriched in actively transcribed genes, the A1 and A2 sub-compartments. Although the contact map suggests that these two sub-compartments are structurally distinct, both A1 and A2 have similar levels of enrichment for active histone marks and open chromatin (though A2 is slightly more enriched in H3K9Me3), so it is unclear how, or whether, they are also functionally distinct.

Although Hi-C quantifies how often two genome regions are in close proximity, light microscopy shows that the spatial distance between loci varies greatly from cell to cell [37]. Such distances cannot be directly captured by canonical Hi-C, which represents only short distances in a multi-cell superposition. Recently, however, single-cell Hi-C of haploid mouse embryonic stem cells (mESCs) has produced 3D structures of whole genomes (modelled as 100 kb particles), thus revealing realistic spatial snapshots of folded genome conformations for individual nuclei [36, 38, 39]. In these structures, segmented chromosome regions are modelled, with distance restraints, as either spatially adjacent or non-adjacent, and the resulting solved 3D structures (from repeated calculations with random start points) show the vast majority of Hi-C contacts support a single, folded genome conformation. With structures of whole genomes, the chromosomal locations of TF binding sites that are closely positioned in 3D can be identified (subject to the modelled resolution), including for linearly distal DNA segments [36]. Although this does not directly show where actual TF proteins were physically located within these individual cells, the co-localization of the TF sites within the 3D structure are easily investigated. Furthermore, single-cell genome structures also clearly show chromosome territories and define trans-chromosome interactions with an equivalent precision to intra-chromosome interactions. This enables the study of TF co-localization preferences at the interfaces between different chromosomes. This is not possible with the available population Hi-C data, due to the reduced data density (and hence resolution) of the *trans*-contact map. In addition, co-localized TF sites observed from *trans*-chromosomal interactions are free from the influence of linear sequence and can thus serve as a good control.

Hi-C derived proximity data provides a way of studying how the distribution of binding sites along the chromosomes is organized in 3D space, and thus how genome structure correlates with in vivo TF binding. Our analysis begins by comparing 3D proximity to TF site occupancy (a measure of binding), given that this has already been shown to be influenced by DNA sequence motifs, chromatin accessibility, and epigenetic marks [1, 11, 40–42]. Additionally, several studies have predicted that chromosome organization can exert influence on TF binding. For instance, based on Brownian dynamics simulations, Brackley et al. showed a network of loops containing multiple homotypic sites can facilitate TF binding to certain genomic loci [17], and in a set of inferred super-enhancer networks, increased TF binding up to twofold was observed by Malin et al., which was hypothesized to result from groups of enhancers being in spatial proximity [12]. Also, from a dynamics analysis of Sox2 protein binding, Liu et al. observed an increase in binding site association rate when Sox2 sites are clustered together [15]. In this paper, we suggest that TF interaction networks are also spatial networks, i.e., TF-TF interactions are correlated with spatial co-localization of TF binding sites. Our informatics analysis does not aim to discriminate whether the TF-TF interactions shape 3D genome architecture and/or whether the 3D organization affects the frequency of TF-TF interactions. Rather, we show that the 3D spatial organization of chromatin can provide insights into the functioning of gene regulatory networks genome-wide.

Using both canonical, population Hi-C contact maps [24] and genome 3D structures derived from single-cell Hi-C [36], we have investigated the spatial co-localization of TF binding sites on a genome-wide scale, both within and between different types of TF protein binding site. Using 3D structures is very helpful to corroborate the results from population Hi-C as they treat single-cell Hi-C data (which is comparatively sparse) in a completely different manner: the structures do not rely on the statistics of summed contact counts. The structures represent the whole shape of the individual folded genome and so are ideal for investigating interactions at wide sequence separations and *trans*-chromosomal interfaces, i.e., where canonical Hi-C is most sparse, and can show whether an observation is present in the genome conformations of individual cells, and not just a statistical average that results from combining many cells. Also, by comparing two different cell types (lymphoblastoid and ESC), we investigate whether spatial features are general across cell types or relate to lineage-specific transcription. Overall, we show how the spatial organization of TF sites, which have been identified using ChIP-seq, can be used to provide deeper understanding into the relationship between transcriptional regulation and genome architecture. Previously, there has been much effort undertaken to construct TF regulatory networks; linking TFs to their target genes, and TF-TF interaction networks; linking TFs that physically interact with one another. This paper introduces a third type of TF network, the spatial network of co-localized TF binding sites, as revealed by Hi-C.

## Results

ChIP-seq profiles for a total of 37 transcription factors in human lymphoblastoid cells (GM12878) and 22 transcription factors in mouse embryonic stem cells (mESC) were obtained from either ENCODE [10] or publications listed in Additional file 1: Table S1a. This resulted in a list of between 635 and 17,884 likely bound sites for the different lymphoblastoid TFs and between 1117 and 33,890 bound sites for mESC TFs. For the human lymphoblastoid data, 96% of ChIP-seq peaks fell into DNase-I hypersensitive sites (DHS). For the mESCs, 74% of ChIP-seq peaks overlap with DHS.

**Fig. 1:**
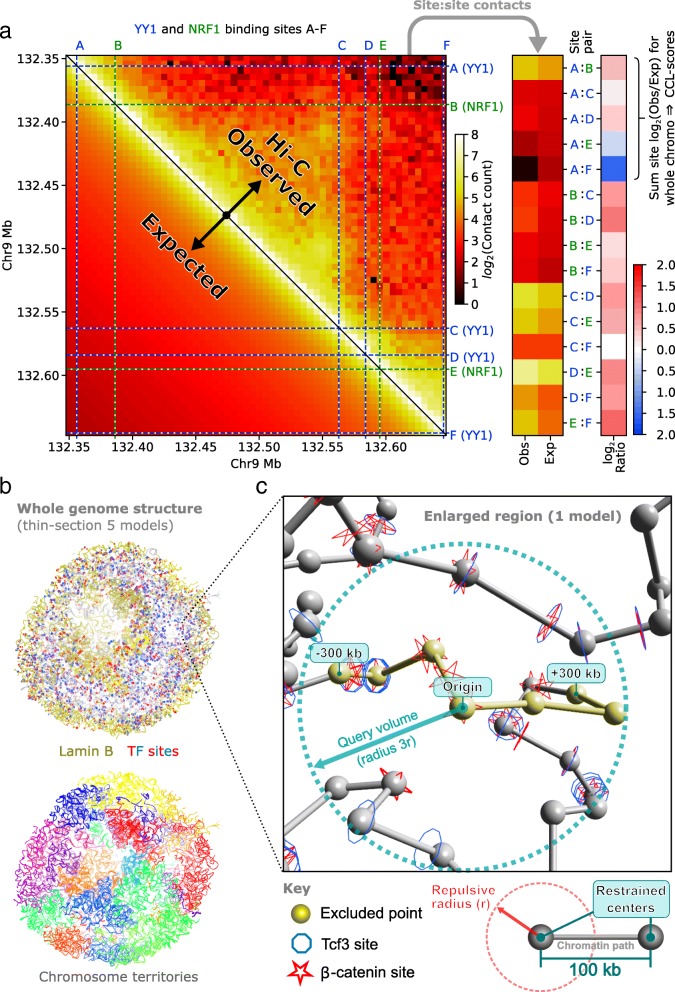
Measuring co-localization of TF sites in Hi-C contact maps and genome structures. **a** A schematic overview of using Hi-C contact data to quantify the spatial co-localization of TF binding sites, both within the same type and between different types. A section of a Hi-C contact map for human chromosome 9 at 5 kb resolution (upper right triangle) showing normalized contact counts of lymphoblastoid GM12878 cells [24] and the corresponding count expectation, given sequence separation (lower left). The illustrated color scale corresponds to the binned contact counts. Illustrative binding sites for two TFs (YY1:blue and NRF1:green), identified by a combination of ChIP-seq and sequence motif scans, are shown as dashed lines. Paired contact possibilities between these sites are shown on the Hi-C map at the intersections of these lines, and the corresponding observed and expected count values for each pair are extracted into separate columns (mid-right panel). For each TF:TF site pair, the log_2_(Observed/Expected) score is shown in the last column (right); it is the summation of these values that is used to calculate the CCL-scores for either a single TF (homotypic) of between different TFs (heterotypic). **b** Studying TF sites in a 3D genome structure calculated from single-cell Hi-C. A genome structure for a single cell, calculated using single-cell Hi-C, provides relative three-dimensional coordinate positions for all chromosomes, here modelled as 100-kb particles. The complete genome is shown as thin sections through the center of five aligned coordinate models and colored according to chromosome identity (bottom). The locations of TF binding sites within these structures can be identified (top). Here, β-catenin sites are shown in red and Tcf3 sites in blue. The data is shown for mouse ESC “Cell1” as published in Stevens et al. [36]. **c** Identifying co-localized TF sites in a genome structure. An enlarged section of one structure model shown in **b** shows the modelled chromatin backbone path (grey/yellow) and illustrates how TF sites within a specified radius of a query point (center of dashed circle) can be identified. The solid spheres represent the restrained points in the middle of 100-kb chromosome regions (so there is also 100 kb between points). The repulsive radius (*r*) used in the structure calculation, to separate the restrained points in 3D space, corresponds to half of the ideal sequential point separation (equivalent to 50 kb). The points that are close in sequence to the query (within 300 kb, either side), which are excluded from its analysis, are shown in yellow

Proximity data was derived from the high-resolution Hi-C of human lymphoblastoid cells (GSE63525 [24]), comprising a total of 4.9 billion chromosomal contacts, and allowed intra-chromosomal (*cis*) Hi-C regions as small as 5 kb to be studied. Combining the Hi-C contact map with predicted genome-wide TF binding sites gave potential intersection points (see Fig. 1a) totalling 1.2 × 10^8^ for homotypic (within the same type) and 3.4 × 10^9^ for heterotypic (between different types) TF site pairs. From the published mouse ESC single-cell genome structures calculated at 100 kb resolution (see Fig. 1b for an example), the six best defined were studied, which derived from 37,000 to 122,000 chromosomal contacts for each cell [36]. Single-cell contact maps were not directly analyzed, rather by mapping potential TF binding sites onto the particle representation of the published structures (illustrated in Fig. 1c), between 1.1 × 10^8^ and 2.3 × 10^8^ heterotypic co-localizations were identified across the range of TFs (within three repulsive radii and excluding sequentially close points). Here, the 100-kb regions that were used to model the genome structures have a different role compared to the smaller binned regions used to study canonical Hi-C contact counts. They are the building blocks for the 3D structure calculation and are restrained to touch one another (or not restrained), according to the comparatively sparse single-cell Hi-C data. The 100-kb region size represents the highest resolution that modelled all single-cell genome structures to high precision (all-particle RMSD < particle radius, as shown in [36]), given the number of contacts available for each cell; finer resolutions result in more unrestrained regions.

### Hi-C contact enrichment as a reporter for TF binding in different genomic contexts

Chromosome ligation frequency in Hi-C can be viewed as an indicator of how likely two pieces of DNA are spatially proximal to each other. Hence, we used a measure of Hi-C contact enrichment above the background expectation (see the “Methods” section, Eq. 1) as a means to quantify the strength of interaction between any pair of TF binding sites. Only *cis* (intra-chromosomal) Hi-C contacts were used in this instance because they are somewhat denser than *trans* (inter-chromosomal) contacts; the probability of observing a *cis* contact has a strong dependence on the sequence separation and *trans* contacts account for ~ 24% of the total, spread over all 253 human chromosome pairings. The contact enrichment was used to create an overall chromatin co-localization score (CCL-score) for each TF site by considering the contact enrichment at the intersection of one site with other TF binding sites within a whole chromosome. This is illustrated in Fig. 1a, Additional file 2: Figure S1a and described by Eq. 1 and accounts for both the innate sensitivity of the Hi-C experiment at different loci and the sequence separation between them. In essence, this score indicates whether a TF site has more or fewer Hi-C contacts to other TF sites than expected, over the entire range of sequence separations. The CCL-score may be applied in the homotypic case, where the sites relate to the same, single TF protein type, and the heterotypic case, where the sites relate to two different TF protein types.

**Fig. 2:**
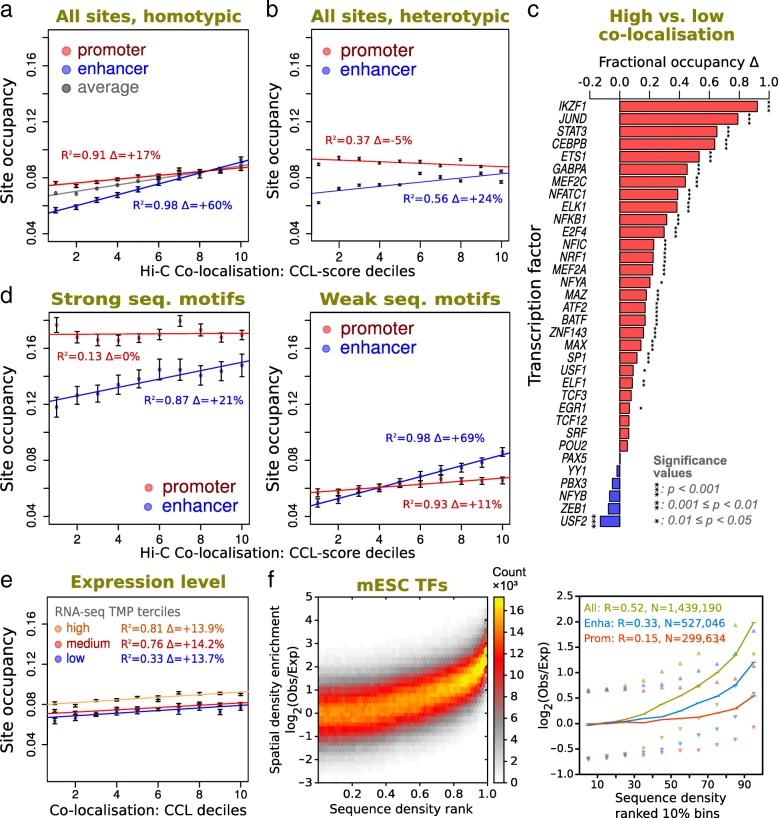
The general correspondence between TF presence and spatial co-localization. **a** Relating TF binding site occupancy with homotypic Hi-C contacts. Correspondences between mean homotypic occupancy and CCL-score (from Hi-C) for all TFs collectively are shown as regression plots and sub-divided according to promoter or enhancer classes (see the “Methods” section). For comparative purposes, the all-site average is shown in grey in the left panel. Accessible sites for different TFs were rank normalized, combined and grouped into ten bins according to CCL-score. Pearson’s *R*^2^ values are shown alongside the percentage change in occupancy change across the CCL range. Error bars indicate standard deviation from resampling. **b** Relating TF binding site occupancy with heterotypic Hi-C contacts. Similar to **a**, but considering interactions between different TF types. For a given site of a specific TF, interactions with all other heterotypic sites were considered collectively to define the integrated heterotypic CCL-score (Eq. 5). Data is separated according to whether sites are found in enhancer regions (blue) or promoter regions (red). All TFs were studied collectively by rank normalization of their heterotypic CCL-score. **c** Occupancy differences between high and low co-localization sites for individual TFs. For each lymphoblastoid TF, the fractional increase in binding site occupancy when comparing the top and bottom terciles of CCL-scores is shown as a bar plot. Stars denote significance level (FDR-adjusted *p* value for a G-test with Williams’ correction). **d** Dissecting the homotypic TF occupancy to Hi-C relationship according to strong and weak sequence motifs. As in **a**, but sub-divided according to promoter or enhancer classes (see the “Methods” section) with either strong (left) or weak (right) DNA sequence motifs, based on motif *p* values obtained from FIMO motif scans [70]. **e** Dissecting the homotypic TF occupancy to Hi-C relationship according to promoter expression. As in **a**, but with gene promoter regions classified according to strength of RNA-seq signal. Accessible sites for different TFs were rank normalized, combined and grouped into ten bins according to CCL-score. Pearson’s *R*^2^ values are shown alongside the percentage change in occupancy change across the CCL range. Error bars indicate standard deviation from resampling. **f** Relating spatial and 1D sequence densities of TF sites in mESC genome structures. The color matrix shows the distribution, for all mESC TFs combined, of the spatial density enrichment (SDE) at different rank-normalized sequence densities. Line plots represent mean values for the distribution of SDE across decile groups of sequential TF density and either represent all TF sites (yellow), enhancers (blue), or promoters (red). Error bars represent standard error of the mean and triangles the 25–75th percentiles. Data shown is for homotypic sites, aggregated for all mESC TFs studied

Given this scoring, we first sought to investigate the correspondence between the homotypic co-localization and measures of TF presence. For the latter, we calculated TF binding site occupancy; the fraction of accessible sites that are associated with ChIP-seq peaks. Given that TF binding may influence and/or be influenced by genome structure, our initial motivation here was to test whether there is a particular linear density of occupied TF sites that has any clear relationship with spatial proximity, which we could then dissect according to genomic features to try to understand the basis for any spatial co-localization. Given a CCL-score for all TF sites (the degree of co-localization to other sites), different sites were ranked for each TF and then combined to study all TFs collectively (see Additional file 2: Figure S1a for details). We found that overall the higher the homotypic co-localization score, the greater the binding site occupancy, as illustrated in Fig. 2a. This is true for sites associated with both promoter and enhancer regions, as identified by chromatin state (determined using histone modification ChIP-seq and DNA accessibility data according to [43]), but the effect is more pronounced for enhancers. Hence, overall, the more homotypic binding sites co-localize, as assayed by Hi-C, the greater the proportion of sites that are bound by their TF. As shown in Fig. 2b, a similar analysis using heterotypic interactions (between different TF types, Eqs. 2 and 5) shows that although the observed relation is weaker in the heterotypic case compared to the homotypic case, a positive correspondence is also present at enhancer regions, but not at promoter regions.

Corresponding analysis of individual TF types showed the positive correspondence between TF binding and homotypic site co-localization is present for most, but not all, TFs separately. For each TF, we grouped binding sites according to their associated homotypic CCL-scores into ternary groups (high, middle, or low), as an indication of how each site is in proximity to other homotypic sites. As illustrated in Fig. 2c, for lymphoblastoid ChIP-seq datasets with > 300 peaks in each score group, 24 out of the 34 TFs have a significant increase in binding site occupancy when comparing the high and low score groups. Dissecting promoter and enhancer regions for each TF yielded similar results (see Additional file 2: Figure S1d and S1e). The behavior of some TFs is clearly different, e.g., for USF2, which is consistent with its negative regulatory domain for enhancer activity [44].

The classification of binding sites at promoters and enhancers may be dissected further according to sequence motif strength or genomic activity. Hence, we initially separated the TF sites according to whether they have weak or strong DNA sequence motifs, as indicated by position weight matrices (Fig. 2d). This showed that the occupancy at weak sites, although less in absolute terms, has both a stronger correlation with spatial co-localization and a larger fold change across the range compared to the strong sites. However, the effect is proportionately small for promoters compared to enhancers. To determine whether the occupancy versus co-localization correlation is linked to genomic activity, as indicated by RNA-seq and epigenetic marks, we dissected promoter and enhancer regions into activity classes (Additional file 2: Figure S1b and S1c, see the “Methods” section for active/inactive, strong/weak definitions) and transcription start sites according to gene expression level (Fig. 2e). Overall, this revealed various degrees of positive correlation, as occupancy increases with co-localization, where enhancers show the greatest difference according to activity. However, although absolute occupancy differs between the activity classes, the proportional changes show little difference.

Next, we sought to further confirm our results by looking at ChIP-seq signal intensity (rather than site occupancy) and accounting for the influence of DNA sequence biases and epigenetic differences at individual sites. Hence, we randomly paired sites that have identical DNA sequences where one binding site is from the top third homotypic co-localization group and the other is from the bottom third co-localization group (i.e., from DNA regions with respectively high and low levels of spatial clustering, as indicated by the CCL-score). In addition, we made sure to assign site pairs that have the same epigenetic marks and chromatin sub-compartments. For each pair of sites, we then compared the intensity of peak regions in ChIP-seq data, as indicated by ChIP-seq SignalValue (a measure for read enrichment in peak regions used by ENCODE [10]). As illustrated in Additional file 2: Figure S2, we observed a significant SignalValue increase in the high co-localization group (Wilcoxon signed rank test, *p* = 1.3 × 10^−8^). Out of the 16 TFs with sufficient data for analysis, ten showed a significant SignalValue increase within the high co-localization group (Wilcoxon signed rank test, *p* < 0.05), while only one of them showed decreased SignalValue (USF2). This independent measure of ChIP-seq SignalValue further confirms more TF binding is associated with higher homotypic site co-localization, even when we control for DNA sequence, epigenetic marks and chromatin sub-compartments.

We next performed an analysis of single-cell mESC genome structures [36]. Here, the sparse single-cell contacts have been used as distance restraints to fold a particle-on-string representation of the chromosome backbone paths. The structures show a clear relationship between TF binding and spatial proximity to sites of the same type. As shown in Fig. 2f, there is a positive correlation between the linear density of bound TF sites (assayed by ChIP-seq over many cells) and the 3D, spatial density enrichment (SDE) of sites, which is assayed in individual cells and specifically excludes comparing sites that are close in sequence (≤ 300 kb). Also, this relationship is somewhat stronger for enhancer-associated sites compared to promoter-associated sites.

Similar trends are observed for individual TFs, and comparative DNA-binding proteins, as illustrated in Additional file 2: Figure S3. Interestingly, the clearest trends are seen not only for several TFs but also for the CTFC, Smc1a, and Smc3 proteins, which have biological roles involving the spatial association of chromatin, e.g., via loop formation [26, 45, 46]. Here, even the linearly sparse sites are associated with a degree of co-localization. The spatial densities of many TFs correlate well at only the highest quantiles of linear site density, e.g., for Nanog, the strongest trend is seen for the top 30%, suggesting a threshold for spatial clustering. Dissecting binding sites within the mESC genome structures according to whether they are close to enhancer marks (H3K4me1 and not H3K4me3) or active promoter sites (transcriptional start sites (TSS) with H3K4me3 and not H3K4me1) also reveals some interesting behaviors (see Additional file 2: Figure S3). Although some TFs are notably more common at either enhancers or promoters, for the TFs which are numerous at both (like Med12, Tcfcp2l1, Esrrb, etc.), the trend is for the spatial relation to be stronger at the enhancer-associated sites.

### Hi-C contact maps and single-cell Hi-C structures show two interaction groups of heterotypic TFs

While initial analyses mainly focused on the co-localization of binding sites for individual TFs, we next investigated whether there was any notable co-localization between binding sites of different TFs and whether these may be attributable to particular TF-TF interactions. We investigated clustering between all possible TF pairs by expressing the Hi-C co-localization scores for each pair as an aggregated contact enrichment value, covering all binding sites, representing whether the pair has more or less co-localization than expected (see Eq. 4). Accordingly, we sought to determine if the clustering between different TFs is significant and whether any groups of TFs can be observed that are similarly co-localized. It should be noted that, because co-localization scores are not symmetric measures (i.e., generally CCL-score for A → B ≠ B → A), the aggregate pair enrichment value is also not symmetric.

**Fig. 3:**
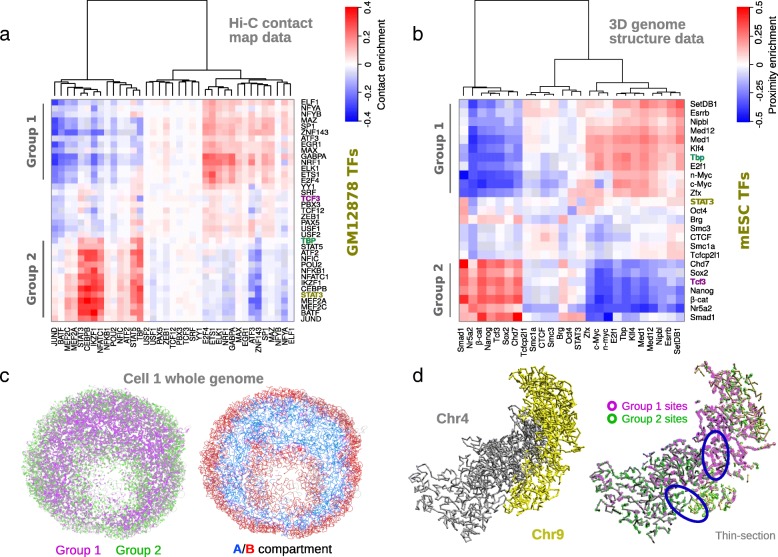
Identification of heterotypic TF co-localization groups. **a** Co-localization enrichment between different TFs in human GM12878 cells. CE values between different lymphoblastoid TF pairs are shown as a color matrix. Colors indicate CE value, where red or blue represents higher or lower than expected contact frequency respectively. Ward’s method [79], using the distance measure in Eq. 5, was used to define row and column orders. Alternative clustering, using Euclidean distances with Wards’ method, is shown in Additional file 2: Figure S4a. The two major sub-network groups that become apparent are labelled at the left. **b** Co-localization enrichment between different TFs in mESC genome structures. Structural proximity enrichment (PE) values between the different mESC TF pairs are shown as a color matrix. Colors indicate PE values; enrichment/depletion of spatially co-localized binding sites compared to the random expectation, where red or blue represents higher or lower than expected co-localization respectively. Data is shown for the six best-defined structures in Stevens et al. [36] combined. Row and column order was determined by using hierarchical clustering based on Wards’ method. The two major sub-network groups that become apparent are labelled at the left. **c** 3D genome distributions of group 1/2 TF sites. Locations of TF binding sites in group 1 and group 2 are shown as purple and green circles respectively and superimposed upon a thin section of a whole genome structure (left). The same view is also shown with the A and B chromosome compartments colored red and blue respectively (right). The data shown is “Cell1” from Stevens et al. [36]; modelled at 100-kb particle resolution using single-cell Hi-C contacts from mESCs. **d** 3D distributions of group 1/2 sites in Chr4 and Chr9. Chromosomes 4 and 9 shown in isolation, taken from the structure shown in **c**. TF binding sites in the group 1 and group 2 groups are shown as green and purple circles respectively

**Fig. 4:**
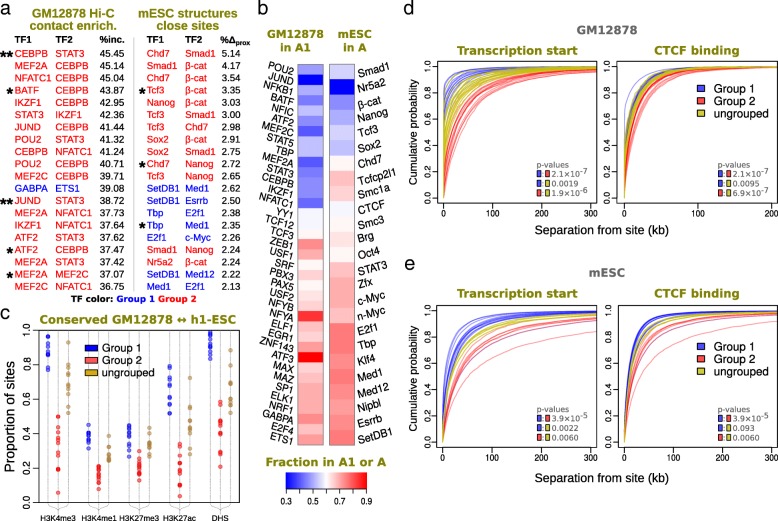
Analysis of heterotypic TF pairs and TF network groups. **a** Top ranked co-localized heterotypic TF pairs*.* The top 20 highly co-localized heterotypic TF pairs identified Hi-C contact maps for GM12878 lymphoblastoid cells (left) and single-cell Hi-C genome structures of mESCs (right). Pairs are ranked by deviation above the random expectation, as described in the “Methods” section. Asterisks represent TF pairings previously identified in the literature and a double asterisk specifically in Wang et al. [89]. See Additional file 1: Table S3 and Table S4 for full ranked lists of scores and significance values. **b** Enrichment of TFs in chromosome compartments. Fractions of TF binding sites in the A1 sub-compartment (lymphoblastoid cells, left) or A compartment (mESCs, right) compared to the total in A1 + A1 or A + B, respectively. TFs are shown in the hierarchical cluster order of Fig. 3. **c** Conservation of TF epigenetic marks between GM12878 and h1-ESC. For various histone mark profiles or DHS profiles, each point represents the proportion of binding sites, genome-wide for each TF, that have a consistent profile between GM12878 and human ESCs. TFs are separated and color-coded according to sub-network group 1 (blue), group 2 (red) or otherwise ungrouped (yellow). **d** Sequence separations of lymphoblastoid TF sites to TSS and CTCF sites. Cumulative distributions of absolute sequence separations from lymphoblastoid TF binding sites to TSSs (left) and CTCF binding sites (right) are shown as line plots, with one line for each TF. Ranked data is cumulatively summed and presented as a proportion of the total. The lines are color coded according to whether the TF is found in group 1 (blue), group 2 (red), or otherwise ungrouped (yellow). *p* values were calculated between TF groups using the Wilcoxon ranked-sum test on the mean absolute deviation of signed sequence separations (i.e., either side of the TF site, rather than the absolute values used in the cumulative plots) to TSS and CTCF sites. **e** Sequence separations of mESC TF sites to TSS and CTCF sites. As in **d**, but for mESC TFs: the distributions of sequence separations from mESC TF binding sites to TSS (left) and CTCF binding sites (right). The data shown is for TFs; the CTCF and cohesin components are not included

Hierarchical clustering of the grid of pairwise contact enrichment values (Fig. 3a) for the human lymphoblastoid TFs shows two clear groupings of TFs that have higher than expected co-localization (positive enrichment) within the group and lower than expected co-localization between groups. We refer to these as group 1 and group 2 (Fig. 3a). However, it is clear there are some pairs of TFs (e.g., YY1 and PAX5) which are close to the random, expected values. As illustrated in Fig. 3b, an analogous analysis was performed for the mouse Hi-C genome structures: for well-defined 3D positions, the observed number of ESC TF sites in structural proximity was compared to the number expected at random, thus generating a proximity enrichment score (Eq. 6). In our analysis, we excluded analysis of sequentially adjacent sites within 300 kb (corresponding to 3 backbone regions), to avoid conflating the linear clustering of TF sites with their 3D clustering. The hierarchically clustered matrix of proximity scores for the TF pairs clearly also shows two distinct groups of TFs in ESCs (excluding non-TF proteins like CTCF, cohesin, etc.). Overall, some of the pluripotency factors clustered together more often than expected. Nanog, Sox2, Nr5a2, Smad1, TCF3, and β-catenin together with Chd7, a chromatin remodeller, formed a highly co-localized group which we termed ESC group 2; while Klf4 and Esrrb, two naïve pluripotency factors, are within ESC group 1 together with mediator complex components and Myc. We numbered the sub-network groups in this way to match groups in the lymphoblastoid Hi-C data according to transcription start site proximity (discussed below, Fig. 4), i.e., not according to TF members or their roles. Indeed, several orthologous TFs (TCF3, TBP, STAT3) are common to both human lymphoblastoid and mouse ESC analyses, but are found in different groups.

Using only the *trans*-chromosomal contact points (see Additional file 2: Figure S4b), where there can be no influence from the sequential clustering of TF sites, also shows almost identical grouping of ESC TFs, thus confirming that the heterotypic groups are robust whole-genome phenomena. However, in the *trans* case, the proximity enrichment scores for CTCF, Smc1a, and Smc3 (the latter two being part of the cohesin complex), which are likely involved with TAD and loop formation, show no enrichment, unlike in the general case. This strongly suggests that these structural proteins interact only in *cis*, i.e., within the same chromosome and not between different chromosomes. The two ESC proximity groups are also clear in each of the six single-cell genome structures (Additional file 2: Figure S5), demonstrating that these TF binding site interactions are likely consistent across all similar (G1 phase) cells. Superposition of TF sites from ESC group 1 and ESC group 2 onto the whole genome structures (Fig. 3c, d) reveals how the global arrangement reflects biases for either the A or B chromatin compartment and how the two networks are somewhat segregated in 3D. However, their region of overlap is fairly diffuse compared to the A/B boundary. Closer inspection of the modelled chromatin path, e.g., at the interface between chromosomes 4 and 9 in Fig. 3d, shows that although there is a clear linear clustering of each group’s sites along the sequence, the folded structure of the genome nonetheless brings together sequentially separated, and inter-chromosomal regions, that are enriched in the same TF group (see circled regions in Fig. 3d).

Within the TF groups, from both lymphoblastoid and ESC cell types, we identified the most significantly co-localising TF pairs, with binding sites that are found in spatial proximity more often than would be expected by chance. Accordingly, the lymphoblastoid group 1/2 pairs were ranked using the enrichment of sites proximal to partner TFs based on the Hi-C contact map, and the ESC group 1/2 pairs were ranked by the enrichment of structurally close binding sites (see Fig. 4a for listings of the top 20 in each case). For the mouse ESC structures, the highly enriched co-localization pairs contain several known TF-TF interaction partners, including Nanog and Chd7 [47] and β-catenin and Tcf3 [48]. For the lymphoblastoid contact data, we can identify 40 TF pairs (out of a total of 780) that have significant co-localization (see Additional file 2: Figure S6e for an example of observed and expected CCL-score distributions). Sixteen of these are associated with group 1 and 24 are associated with group 2 (see Additional file 1: Table S2a), while no cross-group pairs are found. One might expect that two TFs that are more frequently found in the same chromatin compartment would be more likely to have co-localized binding sites. However, the most significant lymphoblastoid pairs co-localize not only across the entire genome, but also *within* A1 and A2 Hi-C sub-compartments [24] (see Additional file 2: Figure S7B and Additional file 1: Table S3a-c). There was insufficient ChIP-seq data to do a corresponding analysis for the B sub-compartments, as they are depleted in actively transcribed genes. The high-confidence pairs have significant overlap with previously reported TF-TF physical interactors: there are at least 10 pairs of known physically interacting TFs that we have independently identified with the lymphoblastoid Hi-C data [49–52] (see asterisks in Fig. 4a and Additional file 1: Table S1b). It is noteworthy that significant self-association (homotypic) can also be identified from this data. Indeed, 15 out of 40 TFs are significantly co-localized, representing greater than sixfold enrichment from the expectation (of approx. 2 out of 40).

Given that the overlap of ChIP-seq peaks is an indicator of co-regulated and interacting DNA-binding proteins [9–11], we next sought to investigate this measure, as compared to expected background values, in the context of the two TF co-localization groups. As illustrated in Additional file 2: Figure S4g and S4h, plotting the ChIP-seq overlap enrichment for TF pairs, in the same order as the hierarchical clustering of Fig. 3, shows correspondence between the co-localization groups and peak overlap: TFs that are proximal in Hi-C are generally enriched with overlapping ChIP-seq peaks. This is especially clear for the lymphoblastoid Hi-C data. There is some similarity for ESC group 2 but the situation is somewhat weaker for ESC group 1. However, the correspondence for ESCs is much stronger when comparing peak overlap to co-localization in the B compartment alone (Additional file 2: Figure S4f). However, some strong features of A compartment co-localization (e.g., Smad1:Nr5a2 being depleted) also show though in the ChIP-seq overlap. In general however, there are some notable differences between the ChIP-seq overlap and 3D co-localization analyses. For instance, it is clear that TFs which are members of the same multi-protein complex (e.g., NFYA/B, USF1/2) or paralogues (e.g., MEF2A/C, c/n-Myc) have strongly overlapping peaks, i.e., they bind to linearly close sequences. While these pairs are adjacent in the co-localization matrix (Fig. 3a), showing they are present in a similar 3D context, they are not the most strongly co-localized by 3D conformation (i.e., at sequence separations much larger that the ChIP-seq peak width). By contrast, SetDb1, which does not have much ChIP-seq peak overlap with either group, shows enriched spatial co-localization with group 1 TFs.

### Lymphoblastoid TF sub-networks show binding biases for chromosome sub-compartments

For most cell types, it is now clear that chromosomes are partitioned into A and B compartments. In the high-resolution lymphoblastoid Hi-C dataset [24] studied here, chromosome sub-compartments A1 and A2 have also been observed. Looking at the pairs of lymphoblastoid TFs that have significant co-localization, we found that most pairs of TFs are either enriched within the A1 or A2 chromosome sub-compartment. Furthermore, when plotting the whole range of binding site enrichments for all TFs in A1 versus A2, it seems that those previously in group 1 are more likely to be enriched in A1, while TFs from group 2 are enriched in A2 (Fig. 4b). To further confirm this, we investigated sequence-matched binding sites in terms of site occupancy and ChIP-seq SignalValue. Here, looking at TF sites with identical sequences, and controlling for epigenetic features, allowed us to separate the influence of the sub-compartments from effects due to sequence affinity. We observed that both occupancy and ChIP-seq SignalValues are generally stronger in the A1 sub-compartment for group 1. Similarly, the values are stronger in A2 for group 2 (Additional file 2: Figure S6g).

Since there are clear differences in TF binding between the A1 and A2 sub-compartments, we investigated whether this was sufficient to account for the presence of two distinct TF spatial networks. Hence, to determine whether the TFs were co-localized within each sub-compartment, a similar analysis to Fig. 3a was performed, but dissected according to sub-compartment. Surprisingly, the two co-localization sub-networks reoccurred in almost the same manner in both of the analyses done independently for both A1 and A2 sub-compartments (Additional file 2: Figure S4c and S4d) and no TF swapped cluster in either analysis (Additional file 1: Table S2b). Similarly, in mESCs, we could also recover ESC group 1 and ESC group 2 within either A or B chromosome compartments (Additional file 2: Figure S4e and S4f). Thus, in both cell types, the two proximity groups are not merely derived from (sub-)compartment organization, though there are clear abundance biases.

### TF spatial sub-networks are closely related to tissue specificity

Given that the presence of TFs is a key determinant of tissue type, we next sought to investigate whether the two sub-network groups of TFs have any tissue-specific characteristics. Histone marks and genome accessibility are features that can be either inherited or modified in the process of lineage specification. Hence, we investigated genomic marks located at TF sites in two different cell types for the same species; the makers would be conserved if the sites were employed in similar regulatory contexts. As illustrated in Fig. 4c, comparing genomic markers at TF sites in human lymphoblastoid with markers in human ESCs clearly shows that group 1 is the more functionally conserved class. The conservation of different histone marks including H3K4me1, H3K4me3, H3K27Ac, H3K27Me3, and DHS sites is significantly higher in lymphoblastoid group 1 members compared to group 2 (*p* = 9 × 10^−8^, Wilcoxon rank sum test), with unallocated TFs having intermediate values. Also, the group members’ functional annotations indicate that group 2 has a role more closely related to lineage-specific functions than group 1. An analysis of Gene Ontology (GO) terms [53, 54] for the TFs in each group (see Additional file 1: Table S2c for *p* values) shows group 2 is enriched in lymphocyte activation, intracellular signal transduction (specifically JAK-STAT cascade) and cellular defence response. By contrast, group 1 shows little enrichment of cell-type-specific pathways, other than general transcription activation, response to oxygen-containing compounds and cellular response to organonitrogen compounds, which suggest constitutive roles (group 2 is also enriched in the above two responses, albeit to a smaller extent). Furthermore, the different biases of the two proximity groups towards A1 or A2 sub-compartments hint at a developmental role for the A2 Hi-C sub-compartment.

A corresponding analysis of the TF groups from mouse genome structures shows that ESC group 2 is enriched in mesodermal and endodermal cell fate specification, Wnt signalling pathway and response to lipids, while both of the sub-networks show GO term enrichment towards stem cell population maintenance. Thus, ESC group 2 shows analogy to group 2 in lymphoblastoid cells, in the sense that it might be more involved in cell lineage specification compared to group 1, although the situations are somewhat different given the complement of TFs involved in the maintenance of pluripotency. We were not able to define-sub-compartments in the structural data, but we found that ESC groups 1 and 2 are enriched in the A and B chromosome compartments respectively (Fig. 4b). Nonetheless, the two co-localization groups remain intact within both the A and B compartment separately (Additional file 2: Figure S4).

As illustrated in Fig. 4d for the different TFs, in addition to having different 3D/spatial organizations, lymphoblastoid group 1 and group 2 have different linear relationships to genomic features. Specifically, the distribution of sequence separations clearly shows that group 1 members are closer to the nearest transcription start site (TSS) and nearest CTCF binding site than group 2 members, and the ungrouped TFs are intermediate. An analogous situation is also observed for ESC TF groups, as illustrated in Fig. 4e, and it is this similarity which we have used to number ESC groups so they match an analogous ESC group, despite the TF members and regulatory context being somewhat different in the two cell types. As shown in Additional file 2: Figure S6c and S6d, similar differences are also present for TAD-like domain boundaries [24] (roughly 200 kb in size) and ESC TAD boundaries [32]. However, the results for these boundaries and CTCF sites are perhaps unsurprising, given that both are known to be enriched near transcription start sites.

### Intra- and inter-TF group co-localization segregates according to regulatory differences

**Fig. 5:**
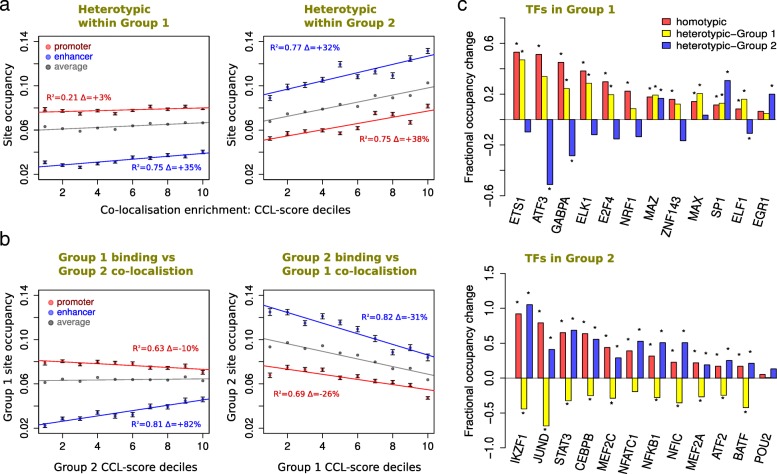
Relationships between heterotypic TF site occupancy and co-localization within and between sub-networks. **a** Relating TF binding site occupancy to the co-localization within proximity sub-networks. Scatter plots with regression lines, separated according to promoter (red) and enhancer (blue) regions, showing the relationship between the mean TF site occupancy and heterotypic co-localization (i.e., between different TF types) within the same co-localization group as measured by CCL-score. Average values for all sites in each of the groups is shown in grey. Binding sites for TFs are rank normalized and grouped into deciles according to the integrated heterotypic CCL-scores within each group. Data is shown separately for TFs from group 1 (left) and group 2 (right). **b** Relating TF binding site occupancy to the co-localization between group 1 and group 2. Similar to **a**, but showing the site occupancy of TFs from one sub-network compared to their co-localization with TFs from the other sub-network. **c** Occupancy differences for high and low TF site co-localization, within and between sub-networks for individual TFs. For each TF within group 1 (top) or group 2 (bottom), the fractional difference in binding site occupancy between the top and the bottom third of CCL-scores plotted as a bar chart. Data is separated into homotypic, intra-, and inter-sub-network co-localization groups. Yellow bars correspond to integrated group 1 CCL-scores, while blue bars correspond to the equivalent measure for group 2; thus, for TFs within group 1, yellow bars represent intra-group co-localization; while for TFs within group 2, blue bars are for intra-sub-network. The effect of homotypic binding site co-localization is also plotted for comparison. The presence of the star above each bar indicates statistical significance (chi-square test with Yates’ correction, *p* < 0.01)

Initially, we showed that homotypic site contacts from Hi-C are correlated with TF presence, and especially so at enhancers (Fig. 2a, d), while in the heterotypic case, the trend is not as clear (Fig. 2b). Given that lymphoblastoid TF group 1 and group 2 are distinct in several ways, we revisited the co-localization versus occupancy analysis for the separate co-localization groups, in both the heterotypic (Fig. 5a) and homotypic situations (Additional file 2: Figure S7a-b). In both cases, the trends are similar: group 2 TF occupancy at enhancers is higher than promoters, with the converse observed for group 1, and the change in occupancy with the CCL-score is generally greater for enhancers than promoters, as we might anticipate. Interestingly, the increase in occupancy with co-localization for promoters in group 2 is much clearer than for group 1. Also, in the heterotypic case, the distinction between groups 1 and 2 is clearer (Fig. 5a). These observations are reinforced by the different separation of group 1 and group 2 TF sites from TSSs (see Fig. 4), i.e., reflecting different structural requirements for sequentially distal elements.

The relationship between the occupancy of group 1 and the heterotypic CCL-scores to group 2 sites (i.e., inter-group spatial clustering) in Fig. 5b shows that clustering with TFs from group 2 increases with the binding of TFs within group 1 for enhancers, but decreases slightly for promoters. This slightly negative trend is perhaps an indication of segregation, i.e., the best occupancy is achieved where the two groups are least proximal. The trend for both enhancers and promoters in group 2, with respect to CCL-scores of group 1, are both clearly negative. Again, this suggests segregation; TF occupancy is highest in group 2 when it is least proximal to group 1. The above trends were further confirmed by analyzing individual TFs within the two sub-networks (summarized in Fig. 5c and see Additional file 2: Figure S7 for further dissection of enhancers and the promoters). For TFs in group 2, there is a consistent negative trend to group 1 proximity, again suggesting general segregation. For group 1 members, the trend seems less clear. However, considering that this does not distinguish between enhancer and promoter regions, the variation could be a mixture of two opposing effects (c.f. Fig. 5b) and further analysis suggests this is indeed the case (Additional file 2: Figure S7c-f).

## Discussion

**Fig. 6:**
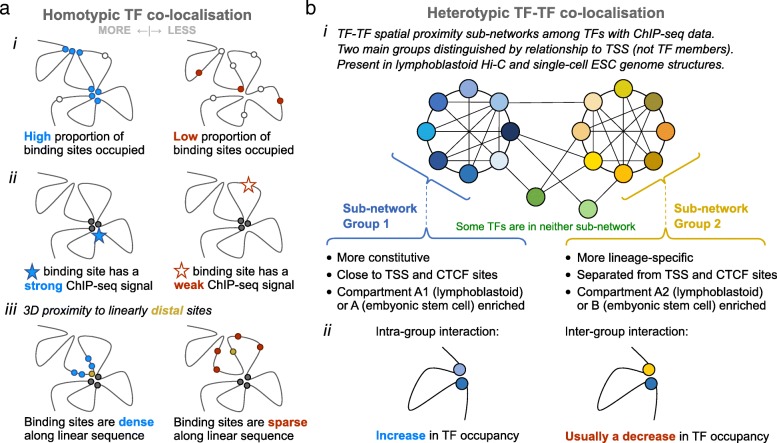
A graphical overview showing the major findings of this study. **a** Measures relating TF presence at binding sites to spatial co-localization. Using Hi-C contacts and single-cell genome structures, our study has shown that, in general, homotypic TF binding site co-localization increases as (i) the bound fraction of binding sites (occupancy) increases, (ii) as the detected ChIP-seq signal for TF sites increases, and (iii) as the linear density of TF sites increases. Also, we observe that these trends are stronger for sequentially distal (i.e., enhancer) and weaker regulatory sites. **b** Grouping of transcription factors into proximity sub-networks. Measuring the degree of co-localization between different TFs, compared to a random background expectation, shows that TFs in both human lymphoblastoid cells and mouse ESCs can be grouped into distinct proximity sub-networks, which appear to correspond to differences in chromatin context and lineage specificity. Furthermore, comparing TF co-localization within and between sub-networks suggests that there is a degree of spatial segregation in TF binding relating to these groups

As summarized in Fig. 6, we have demonstrated that TF-TF interaction networks are reflected in the spatial organization of mammalian genomes. Using both multi-cell Hi-C contact maps and single-cell genome structures, we identified the co-localization of TF binding sites, both in the homotypic and heterotypic cases (Figs. 2 and 3). In the homotypic case, TFs whose binding sites co-localize tend to have higher TF occupancy, even after controlling for DNA sequence and epigenetic factors. Also, clusters of TF binding sites along the linear DNA tend to co-localize with other sequentially separated TF binding sites in 3D structures (Fig. 2f). The mechanism here is perhaps simply that any tendency for TF sites to co-localize in 3D (e.g., via an interaction) will naturally be amplified more if the TF sites are also concentrated in 1D. These trends are further corroborated by our analysis of ChIP-seq peak overlap, which shows that the folded 3D structure often brings together sequentially distal TF sites that also bind close in sequence. It is notable that the 1D site density determined in a multi-cell sample has a clear relationship with 3D co-localization determined in highly variable, single-cell genome structures. This suggests that the spatial clustering of TF binding sites is a consistent feature of genome architecture. Though because each single-cell genome conformation is so different within nuclei [36, 38, 39], this must be achieved with different sets of sites being proximal in each case.

Our observation of a generally stronger relationship between TF binding occupancy and 3D co-localization at (1) binding sites with weak sequence motifs and (2) sites in enhancer regions (defined by chromatin state annotation [43]) suggests that weak binding sites and enhancers need more help from genome organization to achieve TF binding and thus fulfil their regulatory functions. For enhancers, this is perhaps unsurprising, given their known roles in enabling longer-range chromatin contacts and is consistent with the notion that multiple enhancers with shared regulatory functions tend to cluster together to form super-enhancers or transcription factories [29, 55–57]; in this way, more efficient TF binding may be achieved, even for weak motifs, and may be related to the observation that the same chromosome region can show different levels of TF binding in different cell lines. For example, further analysis of ChIP-seq profiles from both human lymphoblastoid and human ESC lines shows that sites with weak sequence motifs have much less conserved binding compared to strong ones (Additional file 2: Figure S8a, Wilcoxon signed rank test, *p* = 3.2 × 10^−5^). This hints that weak TF sites are more sensitive to chromatin organization and could provide a mechanism of lineage-specific control.

These results further support the “crowdsourcing” hypothesis proposed in [12], which suggests that spatially co-localized TF binding sites may lead to higher local concentrations of TFs in certain parts of the nucleus. A contributing factor here could be the association of TF proteins with multiple DNA sites, either directly via multivalent binding or relayed though protein-protein interactions. Interestingly, while the activity state (as assayed by RNA-seq and histone marker ChIP-seq) is clearly linked to the average occupancy of TF sites, it is not obvious that 3D co-localization is facilitating TF binding more in the more active regions. This hints that the 3D co-localization enhancement we observe is strongly based on sequence features, much like TAD/loop boundaries and A/B compartments. Related to this, it is notable that TF co-localization has a strong relationship with chromosome (sub-)compartment organization, given that lymphoblastoid groups 1/2 have strong biases towards A1/A2 sub-compartments [24] and ESC groups 1/2 have biases towards A/B compartments.

When considering co-localization between heterotypic binding sites, we discovered two main clusters of TF-TF associations, both in human and mouse cell lines (see summary in Fig. 6b). In human lymphoblastoid cells, the two distinct TF spatial co-localization groups were defined as TF co-localization network group 1 and group 2 respectively. Analogously, two sub-networks are also present in the mouse genome structures: ESC group 1 and ESC group 2, and these relationships are robust within each single cell, within A and B compartments and also at inter-chromosomal interfaces. Given that analyses were only performed for TFs where ChIP-seq data is available for each cell line, it is possible that a more intricate TF-TF interaction network might be observed if a complete set of mammalian TFs were considered. Nonetheless, the two TF sub-network groups have distinctly different relationships with enhancers (Figs. 5a and Additional file 2: Figure S7), histone marker conservation (Fig. 4c) and transcription start sites (Fig. 4d, e). It is notable that the similarly named co-localization groups in lymphoblastoid cells and ESCs not only represent mostly different TFs, as might be expected for the cell types, but where they do have orthologous TFs in common (in three instances), these are not in equivalent groups. This likely reflects the different regulatory networks, and an example here is TCF3, which is in ESC group 2 but neither main lymphoblastoid group; TCF3 regulates the differentiation of lymphocytes [58] but is involved in the regulation of Wnt signalling in ESCs [48]. This supports a notion that the sub-network groups are 3D/structural observations that differ between pluripotent and differentiated cell types (at least), are closely related to the different A/B and sub-compartmental roles and are not an innate property of individual TFs. We speculate that differences in TF proximity group members between lymphoblastoid and ESC lines may relate to heterochromatin remodelling, especially given the ESC group 2 bias for the (heterochromatic) B compartment.

Enhancer sites have a stronger co-localization relationship within group 2, which is consistent with the notion that group 2 comprises the more differentially regulated TFs. For group 1, the low enhancer occupancy and proportionately small change with Hi-C proximity score are consistent with enhancer-based interactions being less important for these TFs. The observation that lymphoblastoid group 1 and ESC group 1 sites cluster closer to CTCF sites (and thus also TSS) shows that CTCF/cohesin-based TADs/loops [26] may provide a more important contribution to group 1 co-localization. However, it is clear that CTCF/cohesin is not required to observe the co-localization groups per se. Our analysis in ESCs shows that although the co-localization of CTCF and cohesin is absent at chromosome interfaces, the two TF co-localization sub-networks are still robustly present. This is consistent with the global arrangement of chromosomes being at a scale which is larger than can be recapitulated by regional models only involving CTCF and cohesin, such as loop extrusion [26]. Also, a recent study revealed that chromosome compartments are preserved, to a large extent, upon removal of CTCF or cohesin [45, 46]. This leaves an open question of whether TFs could help to establish compartmentalization within chromosome territories and at *trans*-chromosomal interfaces.

TF spatial co-localization sub-networks have a close relationship with TF functional groups and physical protein interactions. Looking at the spatial co-localization of different lymphoblastoid TFs, we have highlighted 40 pairs which are likely to have direct interactions, ten of which have already been identified as physical interaction pairs (see Additional file 1: Table S2d); linking TF spatial networks to protein interaction networks. In terms of function, lymphoblastoid group 2 is enriched in lymphocyte and immune response-related TFs, including NFkB, STAT3, and IKZF1, while group 1 contains mainly constitutively active TFs. Overall, given group 2 is more tissue-specific and is enriched in the A2 sub-compartment, it would be interesting to further investigate the tissue specificity of the A2 versus A1. Indeed, when investigating ChIP-seq peak conservation between lymphoblastoid and ESC data in humans, it seems that sites located within A2 appear to be slightly less conserved compared to A1 (Additional file 2: Figure S8b, *p* = 7.8 × 10^−4^). Enhancers and promoters sometimes show differential behaviors in response to the enrichment of co-localization across TF groups. For instance, co-localization to group 2 sites has a negative influence for group 1 binding in promoter regions, consistent with the sub-networks’ segregation, but a positive one in enhancer regions. This could be linked to the presence of some pioneer-factor-like TFs including JUND, a component of AP1 transcription factor complex [59], and CEBPB [60] within group 2. Those factors have been shown to help open up the chromosome and prime the binding of other TFs, especially in enhancers. That possibly helps to explain why, even given network segregation, the co-localization to group 2 sites still helps the binding of group 1 in enhancer regions.

In mESCs, group 2 is clearly analogous to the corresponding lymphoblastoid group with regard to its members’ more distal site separation from TSS and CTCF sites. Furthermore, ESC group 2 might be more involved in specific cell signalling pathways related to cell differentiation (Wnt and TGFβ signalling). Within the ESC sub-networks, some but not all pluripotency factors cluster together more frequently than expected by chance. Particularly, Nanog, Sox2, and other pluripotency factors within group 2 strongly co-localize with each other and also with Chd7, but showed less than expected co-localization with Klf4 and Esrrb. This is consistent with the functional association between these pluripotency factors [48, 61–63] and also between Chd7, Nanog, and Sox2 [47]. The observation that Klf4 tends to co-localize more with TFs enriched in actively transcribed regions is consistent with the fact that Klf4 prefers to spatially cluster with H3K27ac and H3K4me1 [36]. We note that Klf4 and Esrrb (belonging to ESC group 1) are two factors that abolish expression upon exit from naïve pluripotency [64], while other pluripotency factors within group 2 may also be present in non-naïve states, for example, in epiblast-derived stem cells [8, 62, 65]. Furthermore, β-catenin and Tcf3, two proteins involved in Wnt signalling pathway but with opposing effects, strongly co-localize with each other and also with Nanog etc. This is in line with their promoter co-occupancy together with Nanog and Oct4 [61, 62]. Although Oct4 and Sox2 are in different sub-networks, we nonetheless observe a higher than expected co-localization. Interestingly, an analysis of binding dynamics using microscopy has shown that Sox2 helps the binding of Oct4 [13]. Very few TFs co-localize with partners from both structural sub-networks, though one that does is STAT3, a key factor downstream of LIF and important for naïve pluripotency maintenance [66, 67].

## Conclusions

Transcription factors are regulatory DNA-binding proteins that are critical for the establishment and maintenance of cellular identity within multi-cellular organisms. We demonstrate that the spatial organization and co-localization of TF binding sites can be investigated in a genome-wide context using Hi-C contact data and single-cell genome structures. Our results show that measures of a TF’s presence correlate with its spatial co-localization and hence indicate that TF binding is linked to, and reflected by, the 3D organization of TF sites within the chromosomes. This is especially apparent for weak and linearly distal regulatory elements and suggests a role for the 3D chromosome conformation to allow, and perhaps promote, TF function. We also show that analyzing the spatial co-localization of sites for different TFs provides a way to predict biologically relevant interacting TF-TF pairs. Furthermore, these pairings reveal groups of TFs that occur as distinct proximity sub-networks. These sub-networks are constituted differently in lymphoblastoid and ESC lines and appear to relate to regulatory and lineage-specific differences for the TF groups and may partially explain the chromosome sub-compartments that have been observed in high-resolution Hi-C contact maps.

## Methods

### Data sources

ChIP-seq NarrowPeak profiles for TFs in lymphoblastoid cell line GM12878 were obtained from ENCODE [10]. ChIP-seq profiles for mouse ESCs were obtained from different publications where available, as listed in Additional file 1: Table S1a. We note different studies sometimes used different media to grow mESCs; most used either 2i or serum plus LIF feeder-free media, though a few studies used feeders of MEFs, where noted. To work with consistent genome sequence builds, all human data were converted to hg19 (GRCh37), and mouse to mm10 (GRCh38), using UCSC LiftOver Tool: *https://genome.ucsc.edu/cgi-bin/hgLiftOver*.

### Putative site identification and binding site occupancy

Putative, accessible binding sites were determined genome-wide using a combination of DNA sequence motif searches [68] and DNase-I hypersensitivity (DHS) data [69, 70]. In vivo, a TF does not usually bind to every instance of its DNA sequence motif genome-wide, but rather it binds to a subset of these motifs. It has been proposed that a TF’s binding pattern is not completely dictated by its sequence specificity, but also by the DNA accessibility around the binding site [40, 71]. Given 93% of ChIP-seq peaks of GM12878 used in our study overlap with DHS, a fine mapping of ChIP-seq peak to putative, accessible binding sites genome-wide was used to help to understand the differences between the observed ChIP-seq peaks and predicted sites given DNA sequence information. Potential TF binding sites were first identified by matching their DNA-binding motifs to genomic loci within DNase-I hypersensitive sites (DHS), where the DNA is accessible. To determine which of these sites were occupied in vivo, we analyzed ChIP-seq peaks for corresponding TFs and compared it with those putative sites based on sequence motifs. Specifically, putative TF sites were defined as position weight matrix (PWM) motif matches of a certain transcription factor via FIMO motif scan [70] in DNase-I hypersensitive sites (DHS) (*p* value threshold was set to be 10^−4^ by default) [69, 70] PWM for TF motifs were collected from HOCCOMOCO [72], SwissRegulon [73], and JASPAR [74], where available (see Additional file 1: Table S1b for details). TFs without a suitable PWM motif were removed from subsequent analysis.

After the above filtering, a total of 37 TFs were considered, which both have ChIP-seq profiles and well-defined sequence motifs in GM12878. In GM12878, each ChIP-seq peak from the ENCODE profiles was mapped to the best scoring sequence motif which overlaps with it. In each group of genome regions, occupancy was then defined by the ratio of the number of ChIP-seq identified binding sites and the number of total putative TF binding sites. When plotting occupancy across different groups (e.g., split according to spatial co-localization quantile, as in Figs. 2 and 5), errors were calculated as the standard deviation from resampling 1000 times, each omitting one third of TF sites. Also, the displayed occupancy changes across an axis range (i.e., according to CCL-score) were calculated as percentages relative to the initial value, i.e., *∆* = 100 (end − start)/start.

### Linear TF site density in mESCs

We note in mESCs that many TFs with available ChIP-seq data are pluripotency factors, which can sometimes bind to closed, heterochromatic regions. Hence, putative TP sites defined using the overlap with DHS is not applicable herein. Further, many TFs in mESC are known to have very short, low information content sequence motifs and their binding is more dependent on other partner TFs, for instance, Oct4 and Sox2 [13]. In these cases, using a simple sequence motif scan to define putative sites was not persuasive. Therefore, we adopted a more general measure for TF binding of linear site density defined by ChIP-seq, without considering sequence motif composition. For structural analysis, linear TF site density was calculated in each sequential 100-kb region (i.e., corresponding to a particle in a structural model) as the summation of weighted sites. Here, a weighting was used to avoid boundary effects, e.g., if a TF site lies at the border between two adjacent regions it should contribute equally to both. Accordingly, the weight of each site was calculated as the fraction of a 100-kb segment, centered on the site, which overlaps with each analysis region. In essence, this represents the average, over an analysis region, of counts obtained from a continuous sliding window*.* When showing linear site density for combined TFs (Fig. 2f) values were rank normalized and grouped into quantile bins, as required.

### Genomic marker-based categorization

To account for potential influences from histone marks and chromosome sub-compartment on TF binding, we further grouped ChIP-seq identified binding sites and putative sites in GM12878 according to (1) chromosome sub-compartment annotation reported by [24]; (2) association with H3K4me1, H3K4me3, H3K27ac, H3K27me3, H3K9me2/3, and H3K9ac, detecting whether the center of the site overlaps with those histone marks or not; (3) ENCODE consensus chromatin states [43]; (4) regions within 2000 bp upstream of transcription start sites (TSS) that are also associated with H3K4me3 and H3K27ac, marks for active promoters, and which were further classified as strong (highly active) promoters or weak promoters based on the presence or absence of H3K36me3 within 1000 bp of the TSS (upstream or downstream) where the overlap with H3K36me3 peaks should be at least 300 bp [75, 76]; (5) genomic regions classified as enhancers according to chromatin state and also with H3K4me1, a typical mark for enhancers, which were then further sub-divided into active enhancer regions with H3K27ac, or inactive enhancer regions without H3K27ac, but with H3K27me3 instead.

ChIP-seq BroadPeak profiles (from ENCODE, Broad Institute [10]) for H3K27me3, H3K9me2or3, and H3K36me3 were used due to the dispersive nature of those histone marks, while NarrowPeak profiles were otherwise used to determine if certain sites are associated with specific marks. Genomic regions with ambiguous histone profiles (those overlapped with both H3K27ac and H3K27me3 or both H3K9ac and H3K9me) were removed. In addition, methylated genomic regions [10, 77] were excluded from subsequent analysis to avoid potential influence of DNA methylation on TF binding.

For classification of TF binding sites within the mESC single-cell genome structures, a 2-kb region either side of each binding site was considered. Using the ChIP-seq datasets employed in Stevens et al. [36], enhancer regions required H3K4me1 and no H3K4me3 marks, while promoter regions required a TSS, H3K4me3, and no H3K4me1.

### Quantification of spatial co-localization for homotypic TF sites

#### Chromatin co-localization scores from population Hi-C

We utilized the Hi-C contact score derived from KR normalization, as used for the original high-resolution Hi-C publication [24]*.* In the Hi-C contact map of GM12878 [24], genome loci with low numbers of mappable reads (total associated raw reads less than one third of the median for each chromosome) were removed first to avoid potential biases. Diagonal elements of the Hi-C contact map as well as the adjacent 25-kb regions (corresponding to five bins) either side were excluded to avoid potentially large variations in near-diagonal regions of the contact map [33]. This also enabled us to focus our analysis on the contacts between sequentially distal sites more than 25 kb away.

The normalized intra-chromosome Hi-C contact frequency was used as an indicator of the strength of co-localization between any paired loci in each chromosome. Here, the aim was to establish a metric for each genomic locus that can represent how likely it interacts with any potential binding sites of a specific type in sequentially distal positions. First, the degree of spatial clustering of homotypic binding sites around each genome locus was calculated as follows:

1 $$ {\mathrm{CCL}}_i=\sum \limits_j{\log}_{\mathrm{e}}\left(\frac{{\mathrm{Obs}}_{i,j}}{{\mathrm{Exp}}_{i,j}}\right) $$

Here, CCL is the score for chromatin co-localization; Obs_*i,j*_ refers to the observed Hi-C contact score between each genome locus *i* and each ChIP-seq identified homotypic binding site *j* within the same chromosome; Exp_*i,j*_ is the expected, empirical average of Hi-C contact score given a certain genome distance between *i* and *j* within a certain chromosome. For each genome locus *i* containing a putative site, we found the above score ratios for all pairs of contacts between itself and other ChIP-seq identified sites of the same TF on the same chromosome, where Hi-C contact map yielded sufficient reads (more than 20 mapped raw reads). We then summed the logarithms of those ratios to represent how likely the region of interest can be in contact with homotypic sites.

Given that the average number of Hi-C contacts drops quickly as sequence separation increases, for distal loci, the number of reads can be very low for the smallest bin size of 5 kb. When comparing values, such small counts can lead to proportionately large but somewhat meaningless differences. Therefore, we increased the bin size to be 25 kb when two loci are more than 100 kb apart by merging adjacent bins, and further to 55 kb for loci more than 1 Mb apart.

Homotypic CCL-scores of all genome loci for each TF were rank normalized, i.e., each score was replaced by its fractional rank, and further put into decile groups (10 groups) or grouped into high (top third), mid (middle third) or low (bottom third) levels based on site abundance.

#### Spatial density enrichment from single-cell genome structures

Homotypic spatial co-localization in single-cell genome structures (downloaded from GEO accession GSE80280 [36]) was assessed by calculating the spatial density of each TF site. To give a somewhat continuous measure that could be applied similarly to TFs with quite different total site counts, the spatial density was first calculated based on inter-site 3D distances and then expressed as a log-ratio enrichment, by comparison to the equivalent value for a random/background expectation. Specifically, the radial density *r*_*i*_ for a given TF type was calculated at each structure particle (*i*) from the summation of inverse-cube inter-particle distances *d*_*i*, *j*_ to all other particles (*j*) further than 300 kb apart and weighted by the number of TF sites present within the particle region *n*_*j*_:

$$ {r}_i=\sum \limits_i\frac{n_i}{{d_{ij}}^3} $$

Here, the cube power was chosen to perform a more close-range focused analysis, as compared to the square power used in previous spatial density analyses [36]. Here, the notion is that all structure particles with TF sites have an influence on the density at every other particle, but this influence diminishes rapidly with distance. This can be imagined as the influence of each particle being diluted within a spherical volume.

Equivalent spatial densities were also calculated in the situation where the TF sites are circularly permuted, with random offsets, around the linear chromosome sequence. This was done separately for sites found in A and B compartments, i.e., only permuting within the same compartment type. This procedure ensured that the sequential relationship between TF sites and their A/B compartment distributions was mostly preserved. For each particle (separately), a spatial density was calculated for 100 random permutations and the result was averaged to generate ${r}_i^0$, the null expectation for the radial density at particle *i*.

The enrichment of the observed spatial density compared to the random expectation was then expressed as a log ratio:

$$ {\mathrm{SDE}}_i={\log}_2\left(\frac{r_i^0}{r_i}\right) $$

The distribution of spatial density enrichment (SDE) values for a given TF were only compared to those of other TFs (see Fig. 2e) after first normalising the distributions so that they are similarly centered and scaled. Specifically, a Z-normalization was performed on the 25% of particles that had had lowest *sequential* site density, given that these had values that most closely matched a random normal distribution. Generally, the enrichments had an excellent fit to a bimodal normal, but this was unreliable for TFs with proportionately low site counts.

### Quantification of spatial co-localization for heterotypic TF sites in population H-C

#### Heterotypic chromatin co-localization scores

Similar to the homotypic scores, we also defined heterotypic CCL-scores between two TFs, TF *A* and *B*:

2 $$ \mathrm{Hetero}\ {\mathrm{CCL}}_{i,A,B}=\sum \limits_{j\in B}\log \left(\frac{{\mathrm{Obs}}_{i,j}}{{\mathrm{Exp}}_{i,j}}\right),i\in A $$

This was defined for each site *i* of TF *A*, considering all possible interactions with TF *B* sites on the same chromosome. It should be noted that heterotypic CCL-score is not symmetric, i.e., *CCL*_*A,B*_ was calculated for each site of TF *A*, while *CCL*_*B,A*_ was for each site of TF *B*.

To compare the observed score distribution to the expected, as control, we generated randomized TF *A* sites by permuting binding sites of all available TFs (except TF B) for each chromosome 1000 times, while keeping TF *B* sites fixed. Also, the number of binding sites for each TF on each chromosome was kept the same in the above permutation. This gave the expected score distribution for *CCL*_*A,B*_ and a similar procedure can be used with respect to *CCL*_*B,A*_.

In addition, we also derived a measure of interactions with all other types of binding sites or a subgroup of sites based on heterotypic CCL-score. Simply, assuming that different TFs have additive effects, we defined the integrated heterotypic co-localization score at position *i* for TF A (SumHetCCL_*i*, *AG*_) in respect to sites group *G* with *k* different TFs:

3 $$ {\mathrm{SumHetCCL}}_{i, AG}=\sum \limits_{B\in G}^k{\mathrm{HetCCL}}_{i,A,B} $$

When defining group G to include all TFs excepting A, the score SumHetCCL_*i*, *AG*_ becomes a simple general representation of heterotypic co-localization level around each site, as used in Additional file 2: Figure S1a.

Since chromosome sub-compartments [24] may have potential influence on TF binding, instead of randomly shuffling all binding sites on the same chromosome, we also constructed the control set in the way that binding sites were randomly shuffled within each sub-compartment for each chromosome, which preserves the binding site composition in each sub-compartment. TFs with very low number of ChIP-seq identified binding sites (less than 300) in either A1 or A2 sub-compartment were excluded in further analysis.

The Kullback-Leibler (KL) divergence was used to represent the overall extent to which the observed co-localization distribution differs from the expected co-localization between pairs of TFs, considering all their binding sites. The KL distance (with a sign indicating direction of median shift) between the observed and the expected co-localization score distribution was calculated as follows, which we denote as chromatin contact enrichment (CE) score:

4 $$ {\mathrm{CE}}_{A,B}=\left(\operatorname{sign}\right)\sum \limits_k\left({P}_{\mathrm{obs},k}\bullet \log \left(\frac{P_{\mathrm{obs},k}}{P_{\exp, k}}\right)\right) $$

where *P*_obs, *k*_ is the probability for the CCL_*A*,*B*_ to be *k*, while *P*_exp, *k*_ is the probability for the random expectation. Here, each *k* corresponds to a CCL-score bin of unit width. The sign of the formula depends on the right (+) or left (−) shift of the observed co-localization scores median compared to the control.

We performed Ward’s method and average-linkage hierarchical clustering of TFs based on either squared-Euclidean distance or the following distance measure derived from the CE score:

5 $$ {e}^{-\left(C{E}_{A,B}+{CE}_{B,A}\right)/2} $$

We adopted the R package of “DynamicTreeCut” [78] and used the setting of DynamicTree mode with default parameters to define clusters of TFs based on the dendrogram from the above hierarchical clustering. For comparison, Ward’s clustering [79] was also performed. We noticed that clustering methods gave similar results in almost all cases. The single exception was that, while the distance measure in Eq. 5 for Wards’ method gave rise to well-defined clusters within A2 sub-compartment, average-linkage clustering based on Euclidean distance fails.

### Calling significantly co-localized TF pairs

We called significant co-localization of TF pairs based on the distribution of heterotypic CCL-scores. If two TFs prefer to co-localize, there would be an enrichment of binding sites with high spatial proximity, i.e., higher frequency of sites would associate with high CCL-scores more than expected. The expected control sets were generated in the same way as described heterotypic chromatin co-localization scores. Specifically, for HetCCL_*A*,*B*_, we generated randomized TF A sites by permuting binding sites of all available TFs (except TF B) within each chromosome 1000 times, while TF B sites were kept fixed. Similarly, we can generate the random control for HetCCL_*B*,*A*_ in the same manner. For each TF pair, we calculated empirical *p* values for the observed frequency of sites compared to the randomly shuffled control sets (1000 permutations) in high CCL-score groups (the top 20%, 10%, and 5% in the score distribution were examined). We called significantly co-localized TF pairs by using FDR threshold of 0.05 [80] and requiring significant enrichment of high CCL sites based on both HetCCL_*A*, *B*_ and HetCCL_*B*, *A*_. For comparison, we also identified co-localization pairs within either A1 or A2 sub-compartments, similarly by using randomly permuted control sets within each sub-compartment. Where a simple ranking of lymphoblastoid TF pairs was required (see Fig. 4a and Additional file 1: Table S3), the ranks were assigned according to the percentage increase, when comparing observed to expected, of the number of sites associated with a high level of spatial proximity to partner TFs, i.e., sites that falling into the high HetCCL-score group. For the mESC TFs pairs (see Fig. 4b and Additional file 1: Table S4), ranks were assigned using distances in the genome structures rather than using Hi-C contacts directly. Accordingly, the pairs were ranked by the percentage increase in the number of proximal sites, within three particle radii, in the single-cell genome structures. We annotate this in Fig. 4 as %*∆*_prox_.

### TF binding site conservation between two human cell lines

ChIP-seq NarrowPeak profiles for h1-ES cells were downloaded from ENCODE [10]. To compare binding sites between two human cell lines GM12878 and h1-ESC, ChIP-seq peaks in h1-ESC were matched to their corresponding GM12878 peaks, defined as the peak in h1-ESCs that overlapped with the center of the peak in GM12878 data, such that the center-to-center distance of ChIP-seq peaks in the two cell lines is less than 300 bp. The fraction of mapped ChIP-seq peaks in h1-ESC was used as the indication of binding site conservation level.

### ChIP-seq NarrowPeak SignalValue comparison

To account for the effect of DNA sequence motif composition on TF binding affinity and to seek an independent measure of TF binding abundance other than occupancy defined before, we paired sites with the same sequences and compared their ChIP-seq SignalValues indicated by the ENCODE NarrowPeak caller as a measure of binding strength. We made binding site pairs with exactly the same DNA sequences, and with the same (or no) specific histone marks (including H3K27ac, H3K27me3, H3K4me1, H3K4me3, H3K9me, and H3K9ac) and located within DNase-I hypersensitivity regions without any DNA methylation. Chromosome sub-compartment was also required to be the same where the influence of sub-compartment itself was not investigated. Where a site could be paired with multiple sites of the same category, then all possible pairings were retained. Only ChIP-seq peaks that map to a unique DNA-binding sequence motif overlapping with it from FIMO [70] motif scanning were used in the site pairing procedure. ChIP-seq SignalValues of each TF were rank normalized and represented as fractional values. We then found the differences between the normalized SignalValues for each pair of sites (with high versus low homotypic CCL-score, or within A1 versus A2). As a control, the two binding sites in each pair were randomly shuffled with other sites to obtain the expected distribution of the SignalValue differences.

### TF co-localization in single-cell Hi-C genome structures

Only genome regions corresponding to particles with well-defined 3D coordinates, i.e., an RMSD of < 1 particle radii between 10 structural models, were used throughout our analysis for each cell. We focused our analysis on cell 1 to cell 6 [36], as there are more than 90% of particles containing TF sites meet the above criteria. Two sites are defined to be close to each other only if they appear to be within a certain distance threshold (1.5, 2, or 3 particle radii as is discussed below) in all 10 structural models for each cell. This gave rise to a set of consistently co-localized binding site pairs. (Around 78% to 93% of site pairs in each cell identified only using a single model were retained after defining the consistently co-localized pairs.) To avoid potential local structural effects from model coarseness and parameterization, and also to minimize the effect of sequential binding site sequential clustering, only sites more than three particles away sequentially (corresponding to 300 kb) were considered to identify spatially co-localized site pairs.

The control sets were constructed as follows: sites within A and B compartment were randomly shuffled within each chromosome, while (1) keeping the total site number in A/B compartments within each chromosome the same and (2) keeping the “crowding level” around each binding site the same. The latter criterion was applied due to the fact that certain types of TF sites tend to appear in more crowded regions more often than others, but what we are more interested in is which type of TF it is more likely to be together with, rather than the general level of crowding. Hence, to remove the distortion generated by general level of crowding, we took this into consideration when constructing the random, expected control. To define the crowding level associated with each binding site j, we counted the total number of binding sites *N*_*j*_, regardless of type, that were adjacent to each binding site within a certain 3D distance threshold. Within A or B compartment, we grouped all sites into five equal-sized groups according to the rank of *N*_*j*_, and the random permutation of binding sites was done within each group of similar crowding level 100 times. Further sub-dividing the crowding level groups into 10 or 20 yielded nearly exactly the same control-set results (data not shown). We calculated the enrichment of co-localized sites by comparing the observed and the expected number of spatially adjacent binding site pairs, for each possible combination of two TFs, and thus defined the structural proximity enrichment score (PE) as:

6 $$ \mathrm{PE}=\log \left(\mathrm{Obs}/\mathrm{Exp}\right). $$

For studying TF co-localization, we chose to focus on a small scale and the distance threshold used to generate Fig. 3b is 3 particle radii. Using more conservative threshold, such as 1.5 or 2 radii, gives rise to very similar results (see Additional file 2: Figure S6 for the case of 1.5 radii). The above distance threshold was chosen considering that (1) the average distance between two adjacent particles is 1 radius and (2) the radius of the folded genome structures for each cell is in a range of 20 to 25 particle radii. Given that the RMSD threshold of 1 particle radius sets a lower limit for the distance threshold we can apply, we chose three representative distance thresholds of 1.5, 2, and 3 radii. In addition, if larger thresholds were applied, adjustment for boundary effects near the modelled nuclear surface would be required, similar to [81], which could significantly increase computational complexity. For analyzing enrichment of co-localized pairs between chromosome interfaces, a distance threshold of 1.5 and 2 would not provide sufficient data for several TFs, so only the threshold of 3 particle radii was used in this case.

## Additional files

Additional file 1: **Table S1.** Sources for TF binding data. **Table S2.** Co-localization of lymphoblastoid TF pairs. **Table S3.** Heterotypic co-localization significance for lymphoblastoid TFs. **Table S4.** The most strongly co-localized mESC TF pairs. (XLSX 43 kb)

Additional file 2: **Figure S1.** Further dissection of TF site occupancy and spatial co-localization. **Figure S2.** The relationship between spatial density in mESC genome structures and sequence density for sites of different TFs. **Figure S3.** Differences in ChIP-seq SignalValue between sequence-paired TF sites in high and low co-localization groups. **Figure S4.** Enrichment in co-localization between heterotypic TF pairs. **Figure S5.** Enrichment in co-localization between heterotypic TF pairs in single cells. **Figure S6.** Additional analyses for TF network groups. **Figure S7.** Conservation of TF binding sites between human lymphoblastoid and ES cells. (PDF 4417 kb)

## Abbreviations

CCL: Chromatin co-localization
CE: Contact enrichment
DHS: DNase-I hypersensitive site
ESC: Embryonic stem cell
FDR: False discovery rate
KL: Kullback-Leibler
PE: Proximity enrichment
PWM: Position weight matrix
SDE: Spatial density enrichment
TAD: Topologically associating domain
TF: Transcription factor
TSS: Transcription start site
