# Supplementary material for: Canonical and single-cell Hi-C reveal distinct chromatin interaction sub-networks of mammalian transcription factors
Source: Genome Biol. 2018 Oct 25;19:174. doi: 10.1186/s13059-018-1558-2 (PMC6203279; doi:10.1186/s13059-018-1558-2)
Supplement: Supplementary file 2 — Figure S1. Further dissection of TF site occupancy and spatial co-localization. Figure S2. The relationship between spatial density in mESC genome structures and sequence density for sites of different TFs. Figure S3. Differences in ChIP-seq SignalValue between sequence-paired TF sites in high and low co-localization groups. Figure S4. Enrichment in co-localization between heterotypic TF pairs. Figure S5. Enrichment in co-localization between heterotypic TF pairs in single cells. Figure S6. Additional analyses for TF network groups. Figure S7. Conservation of TF binding sites between human lymphoblastoid and ES cells. (PDF 4417 kb) [file 13059_2018_1558_MOESM2_ESM.pdf]

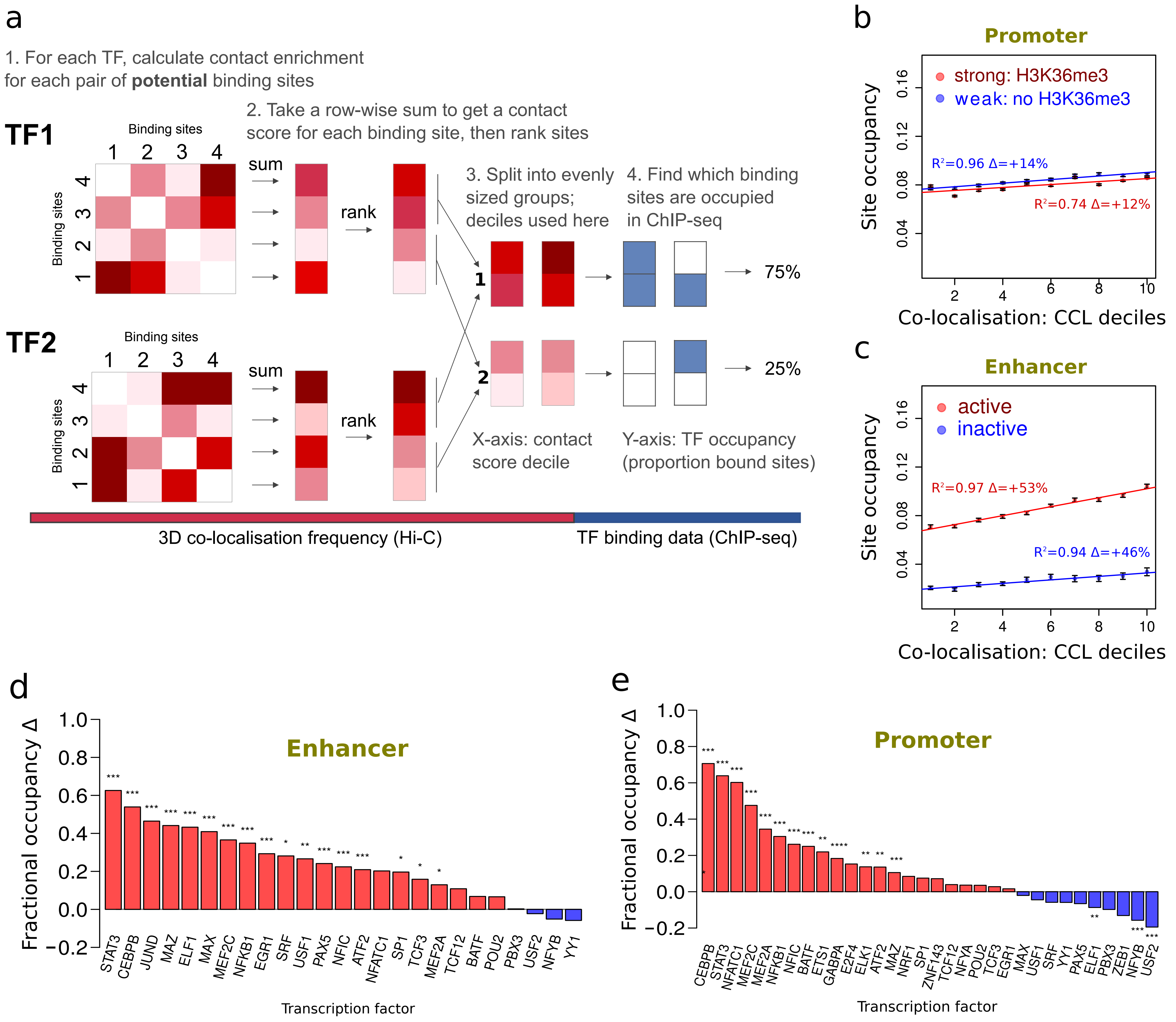

Figure S1. Further dissection of TF site occupancy and spatial-co localisation

a. *Graphical illustration of how co-localization scores are compared with TF site occupancy.*

b. *Dissecting the homotypic TF occupancy to Hi-C relationship according to promoter activity classes.* As in Figure 2b, but with gene promoter regions classified according to strength or activity. See Methods for active/inactive definitions.

c. *Dissecting the homotypic TF occupancy to Hi-C relationship according to enhancer activity classes.* As in **b**, but with enhancer regions sub-divided into strong and weak classes. See Methods for active/inactive definitions.

d. *For TF sites in enhancer regions, differences in occupancy for the most and least co-localized sites, for each lymphoblastoid TF.* The fractional increase in binding site occupancy when comparing the top and bottom terciles of CCL-scores is shown a s a bar plot. The number of stars above the bar plots denote the level of significance (single: $0.01 \le p < 0.05$; double: $0.001 \le p < 0.01$ and triple: $p < 0.001$, using and FDR adjusted p-value for a G-test with Williams' correction).

e. *For TF sites in promoter regions, differences in occupancy for the most and least co-localized sites, for each lymphoblastoid TF.* Presented in the same manner as in sub-panel **b**.

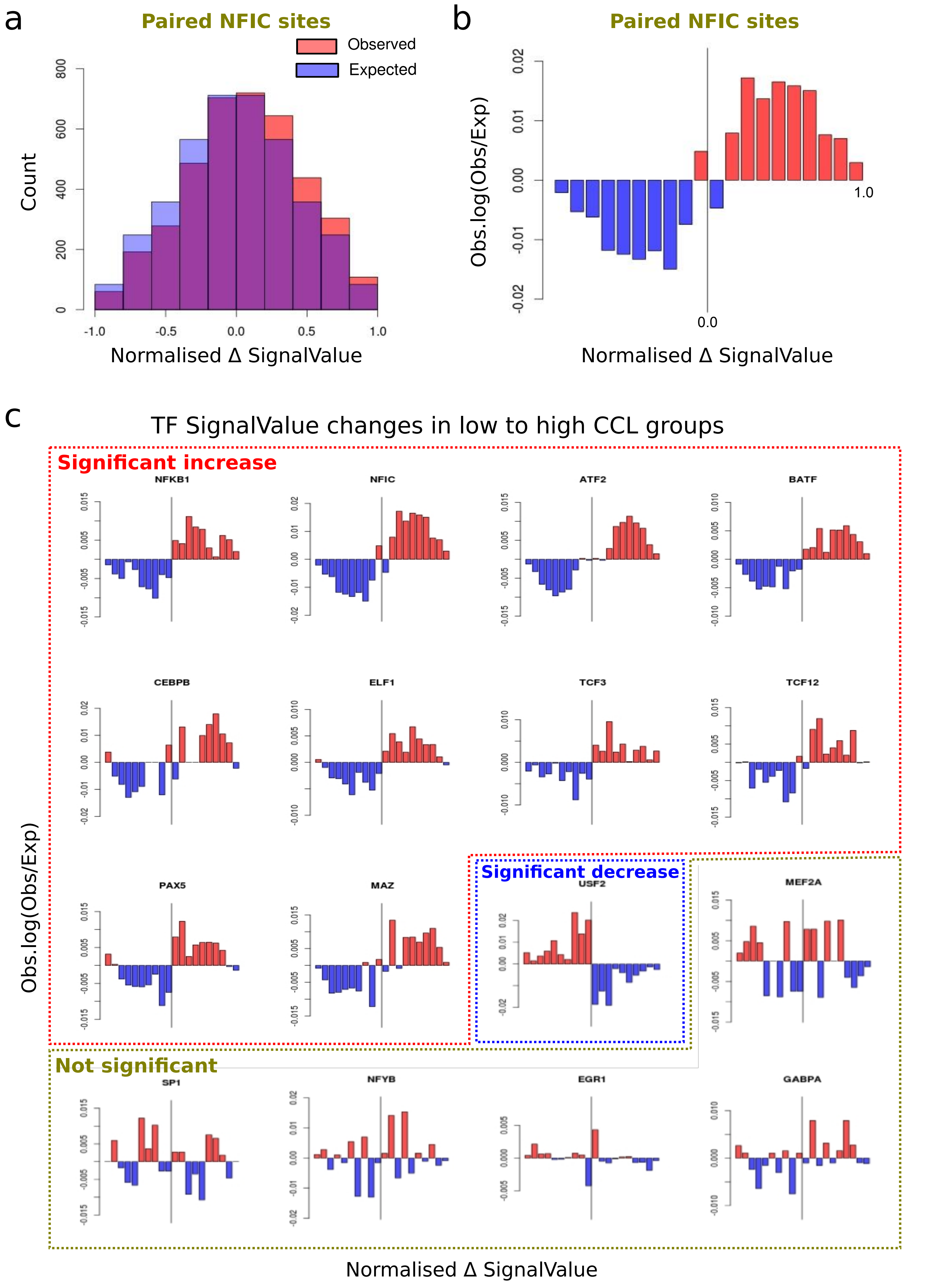

Figure S2. Differences in ChIP-seq SignalValue between sequence-paired TF sites in high and low co-localisation groups
a. *A histogram of observed and expected distributions of the SignalValue difference between high and low co-localisation groups for paired TF sites of an example lymphoblastoid TF NFIC.* Pairs of TFs sites with identical DNA sequences were identified where one site is in the bottom third of CCL-scores (for its TF type) and the other is in the top third of CCL-scores. We further required both site in a pair associate with the same histone marks and locate within the same chromosome sub-compartment. The SignalValue differences for the TF sites were rank normalised between 0 and 1 prior to calculation of differences. The expectation is calculated by randomly permuting sites in each pair.
b. *Enrichment of sequence-pair SignalValue differences for NFIC.* The same type of data as illustrated in a but plotted as a enrichment value to show the differences between observed and expected distributions. A higher than expected count is observed when a binding site with high CCL-score has a higher SignalValue than its paired site with low CCL-score.
c. *Enrichment of sequence-pair SignalValue differences for each lymphoblastoid TF.* As in **b**, but plotted for all lymphoblastoid TFs with sufficient number of paired binding sites (>300). 10 out of 16 TFs are associated with significantly increased SignalValue within the high CCL-score group, 5 have no significant change, while only one has decreased SignalValue.

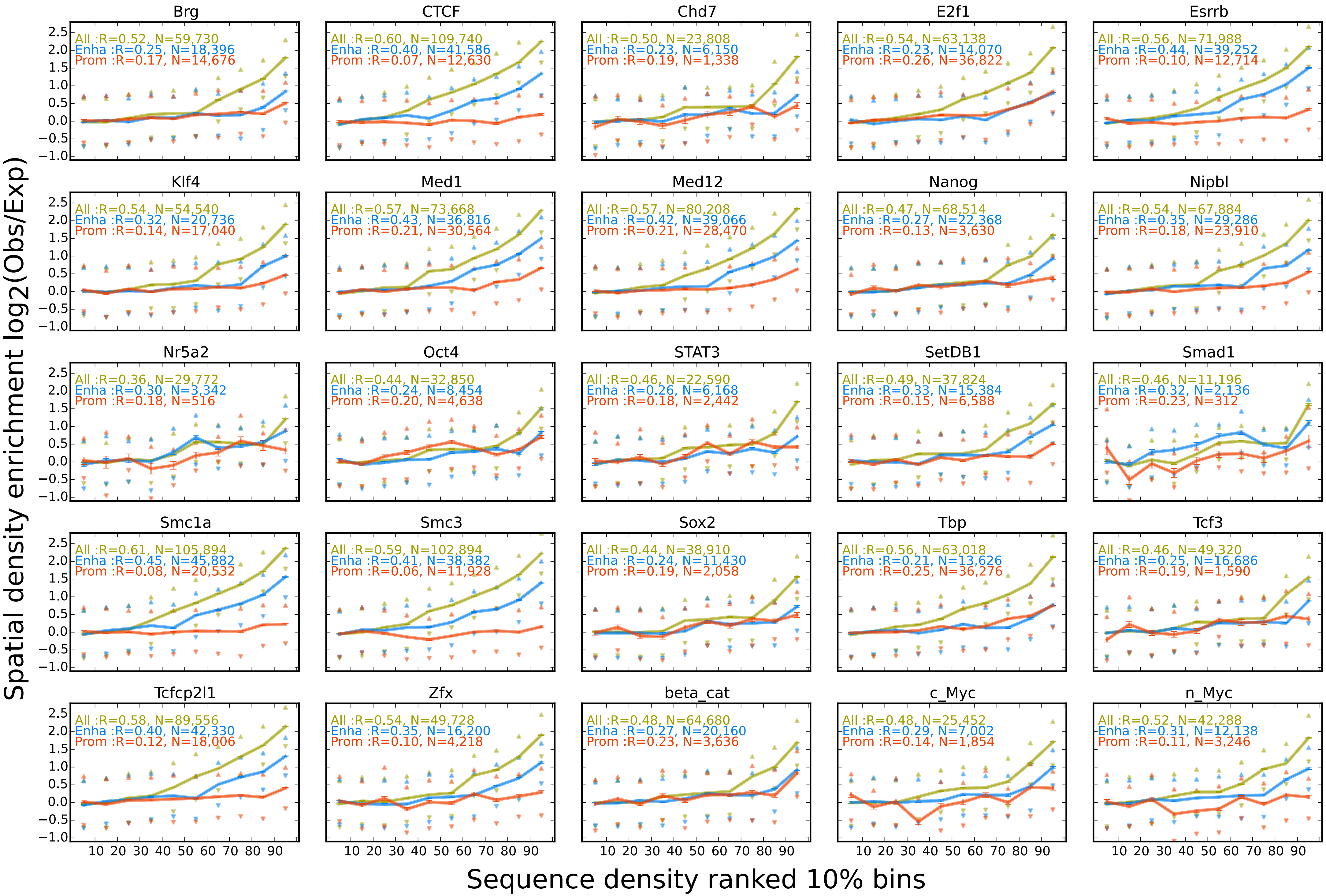

Figure S3. The relationship between spatial density in mESC genome structures and sequence density for sites of different TFs

Line plots represent the mean spatial density enrichment (SDE) for ranked decile groups of sequential TF density. Error bars represent the standard error of the means. Upper and low triangles represent the 25th and 75th percentiles respectively of each decile's distribution. Data is shown for all sites for the TF (yellow), for those sites that fall within 2 kb of enhancer marks (blue) and those within 2 kb of active promoter marks (red). As described in Methods, the SDE is calculated from the for TF site-site distances and compared to a permuted, random hypothesis. The sequential density is calculated as 100 kb sliding window averages, over the 100 kb regions corresponding to structure model particles. Enhancer classification required H3K4me1 and no H3K4me3, while active promoters required a TSS, H3K4me3 and no H3K4me1.

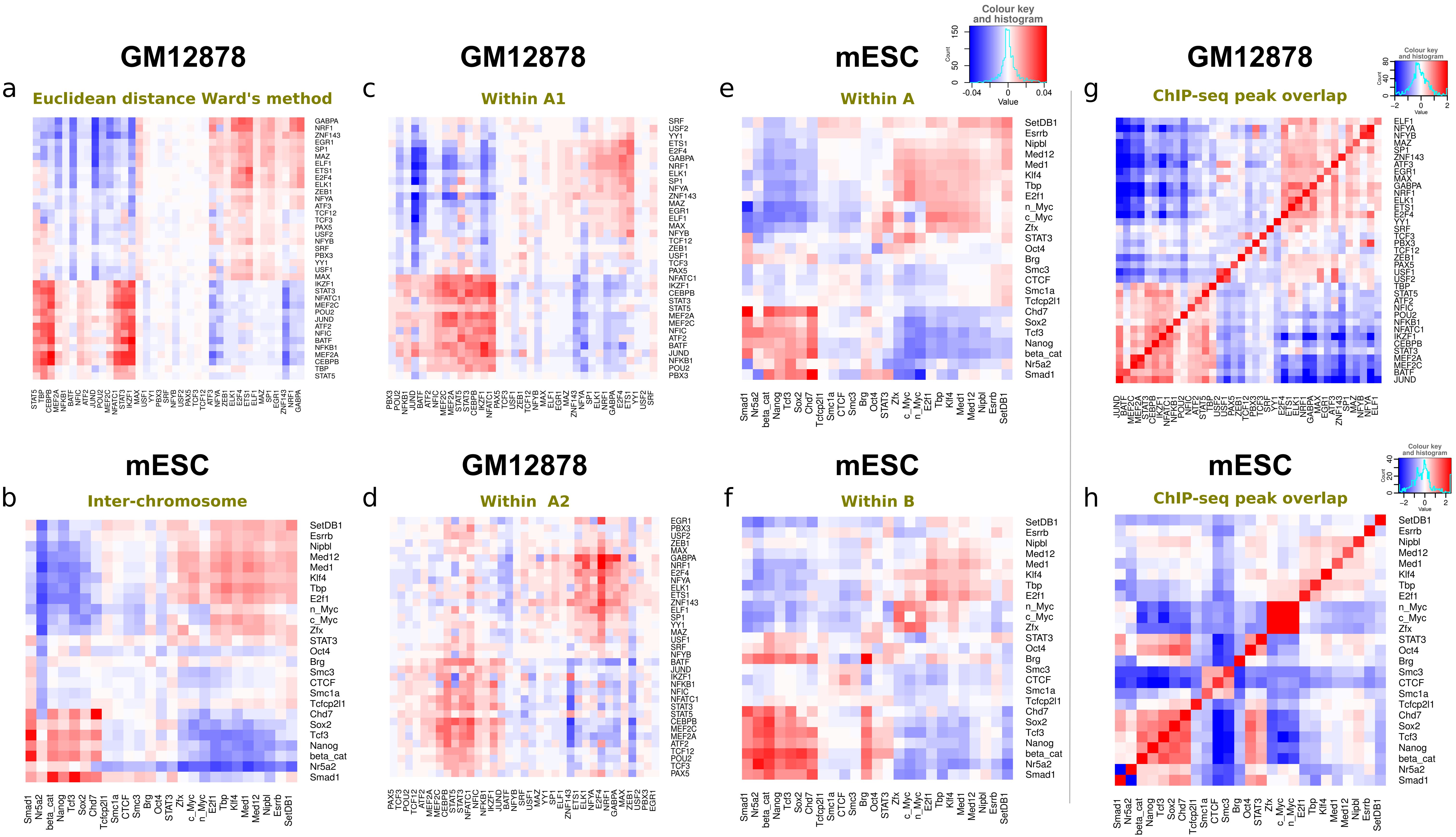

Figure S4. Enrichment in co-localisation between heterotypic TF pairs

a. *Co-localization enrichment between different lymphoblastoid TFs from Hi-C data.* As in Figure 3a, but with a different distance measure for hierarchical clustering. Chromatin contact enrichment (CE) values between the different lymphoblastoid TF pairs are shown as a color matrix. The color scale indicates CE value, where red or blue represents higher or lower than expected contact frequency respectively. Hierarchical clustering, using Ward's method with Euclidean distances, was used to define row and column orders.

b. *Co-localisation enrichment at inter-chromosomal interfaces for TFs in mESC genome structures.*
As in Figure 3b, but showing only data for inter-chromosomal (trans) interfaces in the genome structures. Structural proximity enrichment (PE) values between the different mESC TF pairs are shown as a color matrix. The color scale indicates PE values; enrichment/depletion of spatially co-localized binding sites compared to the random expectation, where red or blue represents higher or lower than expected 3D co-localisation respectively. Data is shown for the six best-defined genome structures in Stevens et al [36]. combined.

c. *Heterotypic lymphoblastoid TF Hi-C co-localization enrichment for the A1 sub-compartment.* As in Figure 3a, but only showing data for TF sites located within the A1 chromatin sub-compartment identified from high-resolution Hi-C data[24].

d. *Heterotypic lymphoblastoid TF Hi-C co-localization enrichment for the A2 sub-compartment* As in Figure 3a, but only showing data for TF sites located within the A2 chromatin sub-compartment.

e. *Mouse ESC TF co-localization enrichment in genome structures for the A chromatin compartment.* As in Figure 3b, but only showing data for TF sites located within the A compartment as defined previously from Hi-C data [36].

f. *Mouse ESC TF co-localization enrichment in genome structures for the B chromatin compartment.* As in Figure 3b, but only showing data for TF sites located within the B compartment.

g. *Overlap of ChIP-seq peaks between lymphoblastoid TF pairs.* Overlap was defined by ChIP-seq peak centres being within 300 bp; the overlapping regions of the peaks themselves was not used due to the large variation in peak widths between different TFs. Colour matrix hues indicate the log ratio between the observed and expected of fraction of ChIP-seq peaks which overlap between the corresponding TF types. The expectation value was calculated by randomly choosing sites for one TF type (and averaging 100 times) while keeping sites for the other TF fixed. The strong diagonal elements represent the perfect overlap of a set of peaks with itself, thus illustrating the upper limit.

h. *Overlap of ChIP-seq peaks between mouse ESC TF pairs.* As in **g**, albeit for a different complement of TFs ith different CHIP-seq data.

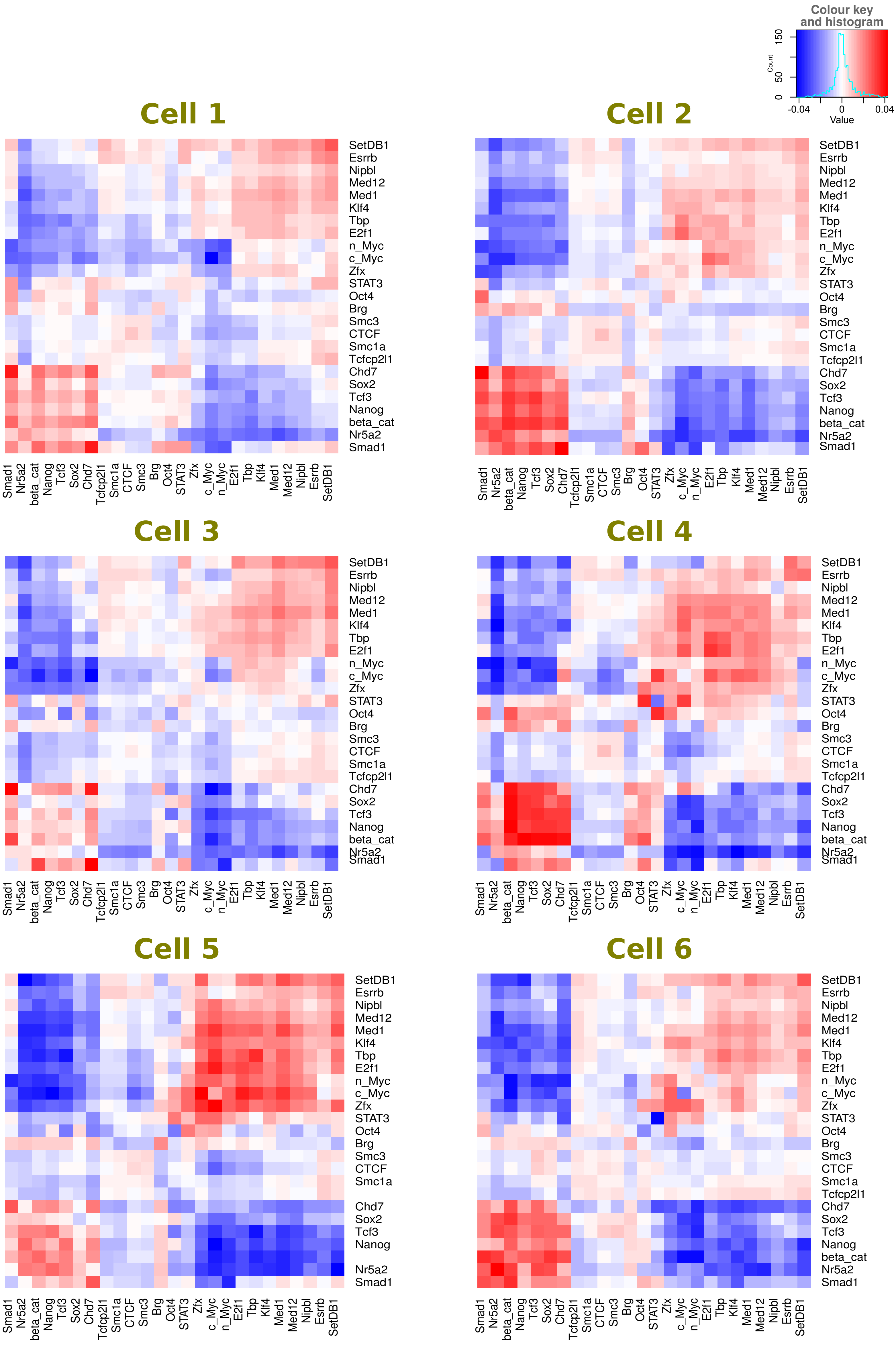

Figure S5. Enrichment in co-localisation between heterotypic TF pairs in single cells
As in Figure 3b, but showing data separately for each of the six, best-defined single-cell genome structures. Structural proximity enrichment (PE) values between the different mESC TF pairs are shown as a color matrix. The color scale indicates PE values; enrichment/depletion of spatially co-localized binding sites compared to the random expectation, where red or blue represents higher or lower than expected 3D co-localisation respectively.

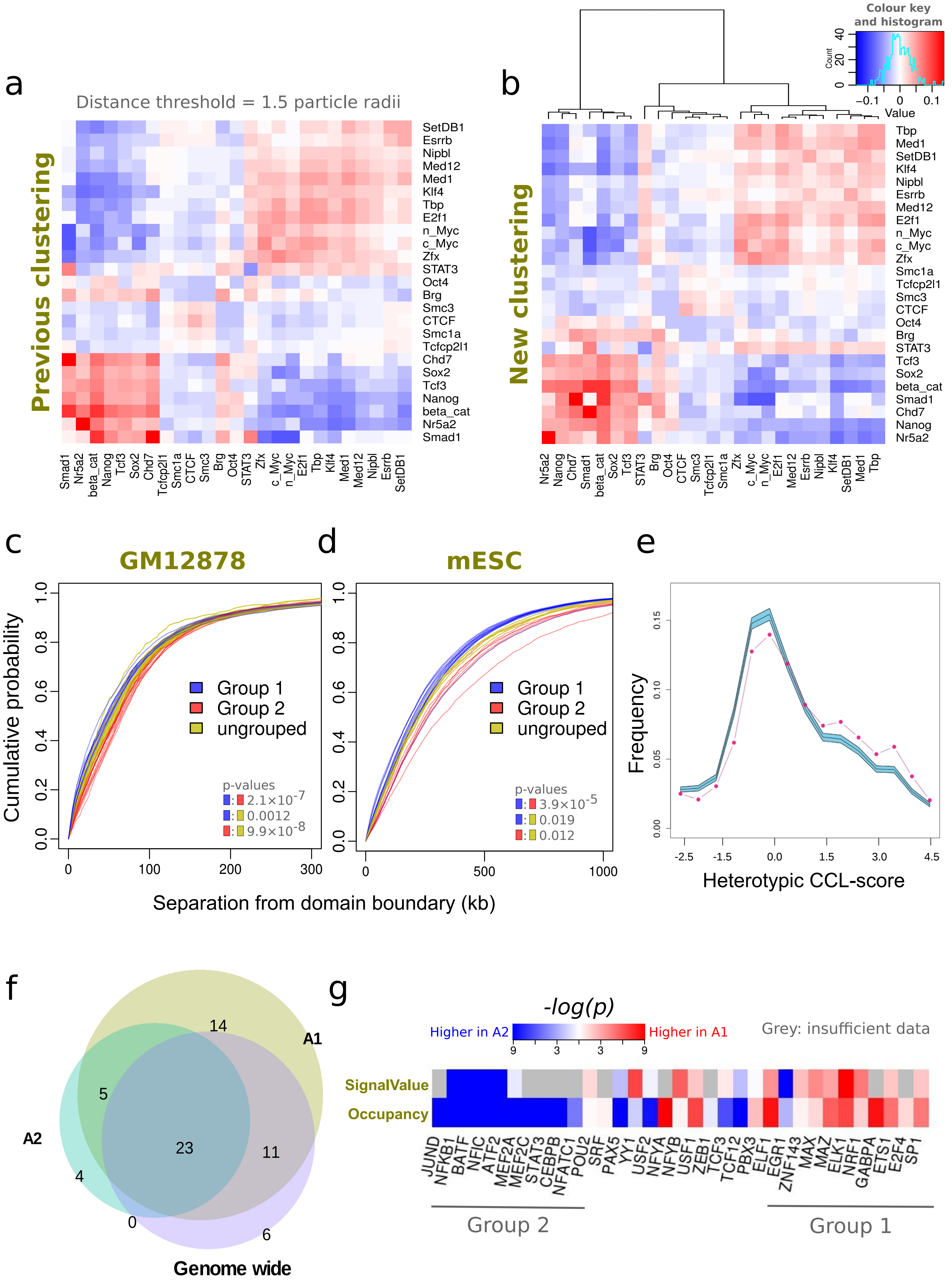

Figure S6. Additional analyses for TF sub-network groups

a. *Mouse ESC TF co-localization enrichment in genome structures using a smaller distance threshold.* As in Figure 3b, but only showing Structural proximity enrichment (PE) values calculated with a distance threshold of 1.5 (rather than 3.0) particle radii. Data is shown in the same row/column order as Figure 3a, i.e. using the original hierarchical cluster order.

b. *Mouse ESC TF co-localization enrichment in genome structures for the B chromatin compartment.* As in Additional File 6: Figure S6a, but with re-done hierarchical clustering

c. *Sequence separations of lymphoblastoid TF binding sites relative to TAD-like domain boundaries.* Cumulative distributions of sequence separations from lymphoblastoid TF binding sites to TAD-like domain boundaries, called using the Arrowhead Algorithm[24], are shown as line plots, with one line for each TF. Ranked data is cumulatively summed and presented as a proportion of the total. The lines are color coded according to whether the TF is found in Group 1 (blue), Group 2 (red) or otherwise ungrouped (yellow). P-values were calculated between TF groups using the Wilcoxon ranked-sum test on the mean absolute deviation of signed sequence separations.

d. Sequence separations of mESC TF binding sites relative to TAD boundaries. The distributions of sequence separations from murine ESC TF binding sites to TAD boundaries[32]. Shown as line plots, with one line for each TF, the lines are color coded according to whether the TF is found in Group 1 (blue), Group 2 (red) or otherwise ungrouped (yellow).

e. *Observed and expected distributions of heterotypic co-localisation scores between SP1 and GABPA.* The observed fractions of sites associated with certain heterotypic CCL-scores are shown for each histogram bin (magenta spots). The expected values (blue) are depicted to show the 5th and 95th percentiles of the distributions, based on the randomized control.

f. *Counts of significantly co-localized lymphoblastoid TF pairs.* A Venn diagram showing the numbers of heterotypic TF pairs that are significantly co-localized within the genome as a whole and/or in the A1 and A2 sub-compartments.

g. *Binding site occupancy differences for individual TFs between chromatin sub-compartments.* Colors indicate the *−log10(p)* of site occupancy differences (bottom row, G-test with Williams' correction) and ChIP-seq SignalValue differences (top row, Wilcoxon signed-rank test for paired sites) between A1 and A2 chromosome sub-compartment for individual TFs.

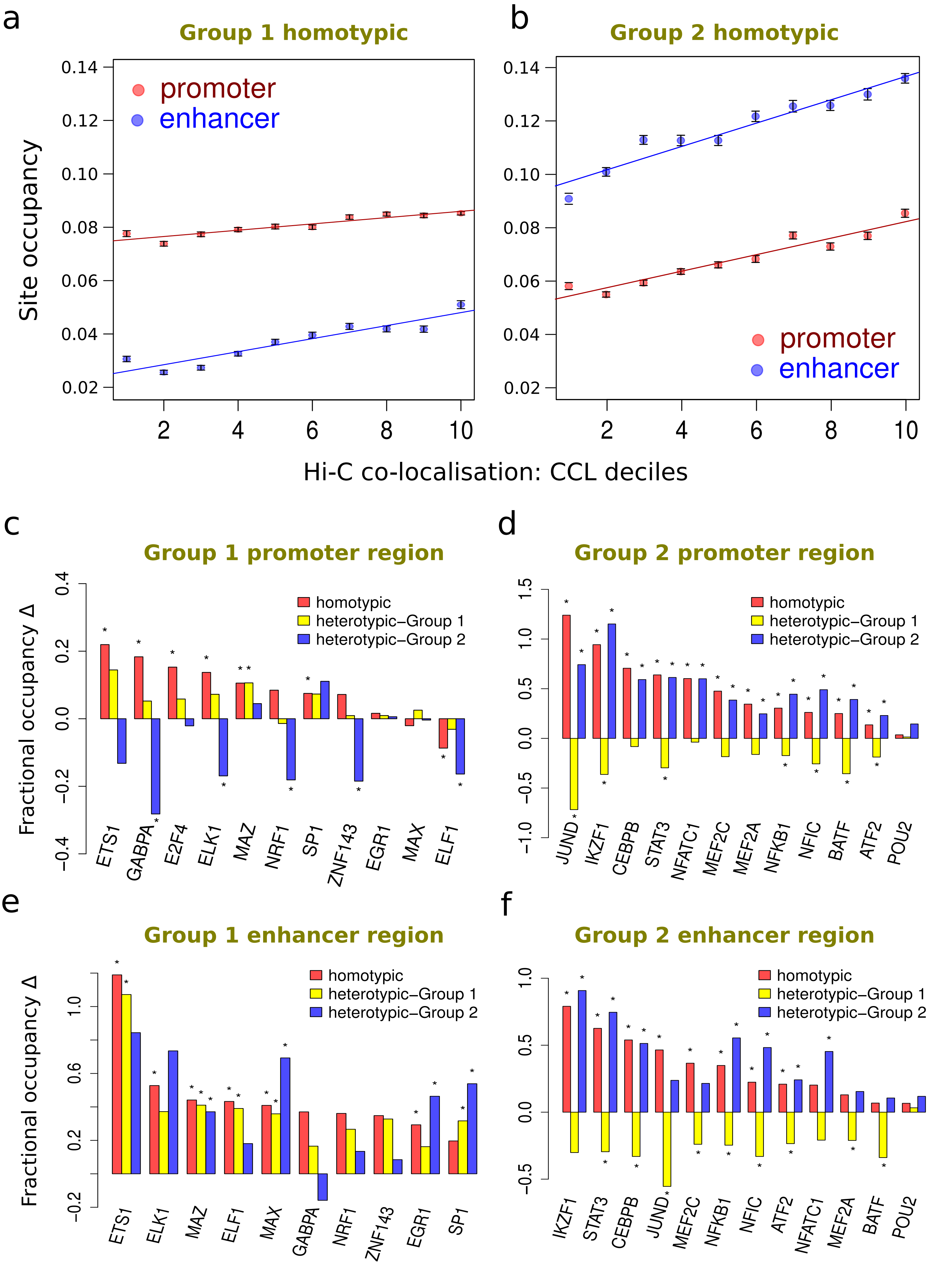

Figure S7. Additional analyses for TF network groups.

a. *Relating TF binding site occupancy to the homotypic co-localisation within Group 1.* As in Figure 5a, but for homotypic sites (rather than heterotypic sites) for TFs in Group 1. Scatter plots with regression lines, separated according to promoter (red) and enhancer (blue) regions, showing the relationship between the mean TF site occupancy and homotypic co-localisation as measured by CCL-score. Binding sites for TFs are rank normalised and grouped into deciles according to the integrated heterotypic CCL-scores

b. *Relating TF binding site occupancy to the homotypic co-localisation within Group 2.* As in **b**, but for for TFs in Group 2.

c. *Occupancy differences for high and low TF site co-localisation, within and between sub-network s for Group 1 sites in promoter regions.* As in Figure 5c, for each TF within Group 1 but selecting only sites in promoter regions, the fractional difference in binding site occupancy between the top and the bottom third of CCL-scores plotted as a bar chart. Data is separated into homotypic, intra- and inter-subnetwork co-localization groups. The presence of the star above each bar indicates statistical significance (Chi-square test with Yates' correction, $p < 0.01$)

d. *Occupancy differences for high and low TF site co-localisation, within and between sub-networks for Group 2 sites in promot er regions.* As in **c**, but for TFs from Group 2.

e. *Occupancy differences for high and low TF site co-localisation, within and between sub-networks for Group 1 sites in enhancer regions.* As in **c**, but for Group 1 sites located in enhancer regions.

f. *Occupancy differences for high and low TF site co-localisation, within and between sub-networks for Group 2 sites in enhancer regions.* As in **e**, but for TFs from Group 2.

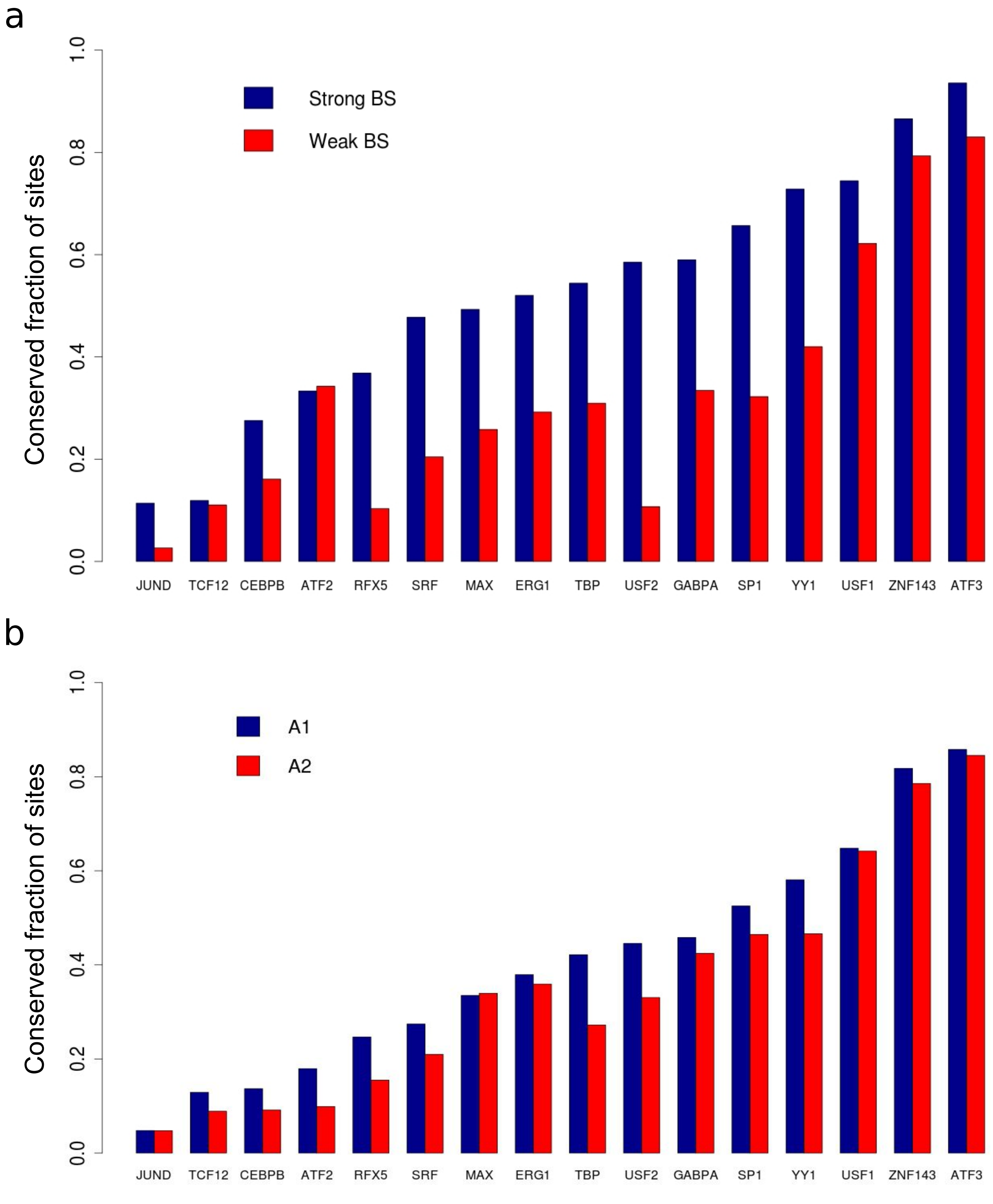

Figure S8. Conservation of TF binding sites between human lymphoblastoid and ES cells

a. *Conservation of strong and weak TF binding sites between cell types.* A bar plot showing the conservation of TF sites between lymphoblastoid and embryonic stem cell types in humans, split according to strong (blue) or weak (red) binding site motifs.

b. *Conservation of A1 and A2 sub-compartment TF binding sites between cell types.* A bar plot showing the conservation of TF sites between lymphoblastoid and embryonic stem cell types in humans, split according to sites found in the A1 (blue) or A2 (red) chromatin sub-compartments, as defined for the high-resolution Hi-C data.
